# Distinct evolutionary trajectories of primary high-grade serous ovarian cancers revealed through spatial mutational profiling

**DOI:** 10.1002/path.4230

**Published:** 2013-08-06

**Authors:** Ali Bashashati, Gavin Ha, Alicia Tone, Jiarui Ding, Leah M Prentice, Andrew Roth, Jamie Rosner, Karey Shumansky, Steve Kalloger, Janine Senz, Winnie Yang, Melissa McConechy, Nataliya Melnyk, Michael Anglesio, Margaret TY Luk, Kane Tse, Thomas Zeng, Richard Moore, Yongjun Zhao, Marco A Marra, Blake Gilks, Stephen Yip, David G Huntsman, Jessica N McAlpine, Sohrab P Shah

**Affiliations:** 1 Department of Molecular Oncology, British Columbia Cancer AgencyVancouver, Canada; 2 Centre for Translational and Applied Genomics, British Columbia Cancer AgencyVancouver, Canada; 3 Department of Computer Science, University of British ColumbiaVancouver, Canada; 4 Department of Anatomical Pathology, Vancouver General HospitalVancouver, Canada; 5 Canada’s Michael Smith Genome Sciences Centre, British Columbia Cancer AgencyVancouver, Canada; 6 Genetic Pathology Evaluation Centre, Vancouver General HospitalVancouver, Canada; 7 Department of Pathology and Laboratory Medicine, University of British ColumbiaVancouver, Canada; 8 Department of Gynecology and Obstetrics, University of British ColumbiaVancouver, Canada

**Keywords:** high-grade serous ovarian cancer, intratumoural heterogeneity, clonal evolution

## Abstract

High-grade serous ovarian cancer (HGSC) is characterized by poor outcome, often attributed to the emergence of treatment-resistant subclones. We sought to measure the degree of genomic diversity within primary, untreated HGSCs to examine the natural state of tumour evolution prior to therapy. We performed exome sequencing, copy number analysis, targeted amplicon deep sequencing and gene expression profiling on 31 spatially and temporally separated HGSC tumour specimens (six patients), including ovarian masses, distant metastases and fallopian tube lesions. We found widespread intratumoural variation in mutation, copy number and gene expression profiles, with key driver alterations in genes present in only a subset of samples (eg *PIK3CA*, *CTNNB1*, *NF1*). On average, only 51.5% of mutations were present in every sample of a given case (range 10.2–91.4%), with *TP53* as the only somatic mutation consistently present in all samples. Complex segmental aneuploidies, such as whole-genome doubling, were present in a subset of samples from the same individual, with divergent copy number changes segregating independently of point mutation acquisition. Reconstruction of evolutionary histories showed one patient with mixed HGSC and endometrioid histology, with common aetiologic origin in the fallopian tube and subsequent selection of different driver mutations in the histologically distinct samples. In this patient, we observed mixed cell populations in the early fallopian tube lesion, indicating that diversity arises at early stages of tumourigenesis. Our results revealed that HGSCs exhibit highly individual evolutionary trajectories and diverse genomic tapestries prior to therapy, exposing an essential biological characteristic to inform future design of personalized therapeutic solutions and investigation of drug-resistance mechanisms.

## Introduction

Ovarian cancer is the leading cause of death due to gynaecological malignancies in the developed world. High-grade serous ovarian cancer (HGSC) represents the most common histology (70%) and is responsible for the majority of advanced-stage cases. Although initially highly responsive to platinum compounds 1, the majority of HGSCs recur and affected women ultimately succumb to their disease.

*TP53* mutation is believed to be the earliest tumourigenic driver event, with presence in nearly all cases 2–3, including pre-invasive serous tubal intraepithelial carcinomas (STICs), suggesting an HGSC aetiology in the fallopian tube 1–9. Compromised homologous recombination due to *BRCA* dysfunction, through inherited germline polymorphism, somatic mutation or epigenetic silencing 3,10, occurs in 50% of HGSC cases. The prevalence of *TP53* mutations and *BRCA* deficiency leads to incompetent DNA repair and likely contributes to chromosomal instability, resulting in severely aberrant karyotypes. Additional recurrent but low-frequency somatic mutations have been reported in *NF1*, *CDK12* and *RB1*; however, in general, *inter*tumoural heterogeneity of mutation profiles is widespread 3.

The concept of *intra*tumoural heterogeneity has long been blamed for treatment failure in ovarian carcinoma and other primary cancers. Rooted in the clonal evolution model of tumour progression, cancers are assumed to originate from a monoclonal composition (the ancestral clone), with subsequent evolution leading to selective expansions of genomically distinct subclones 12 with distinct phenotypic properties 12–16. As such, the divergence of clonal cell populations confers a heterogeneous genomic tapestry with implications for clinical endpoints, including the acquisition of metastatic potential 17–21 and chemotherapeutic resistance 22–23. Models of clonal evolution have been suggested for HGSC through low-resolution genomic profiling 24,25 and serial mutation profiling of primary and recurrence-paired samples 27. However, little is known about the intrinsic diversity of mutational landscapes in primary tumours prior to therapeutic intervention.

Next-generation sequencing technology has enabled the study and quantification of clonal evolution and intratumoural diversity in cancer 20–30, including inference from distributions of digital allelic representation of mutations in single samples 28,29, along with serial 18,20 and regional comparisons of mutation profiles 31. In addition, sequencing of cell-free circulating tumour DNA (ctDNA) extracted from plasma has been demonstrated to be an effective non-invasive tool for monitoring tumour burden 32–33.

Given these advances, we set out to measure precisely the clonal diversity of HGSCs present at diagnosis. Our results show how HGSCs each exhibit unique evolutionary trajectories with widespread regional diversity in mutational, copy number and gene expression landscapes prior to treatment intervention.

## Materials and methods

Six women (Table 1) with histologically diagnosed high-grade serous cancer were included in this study (see supplementary material, Appendix). Tissue was obtained from a total of 31 tumour sites (range 3–10 samples/patient, including two fallopian tube lesions) with matched normal DNA for each patient (Table 1). In addition, for a subset of cases (four patients), plasma (pre-operative and pre-anaesthetic) was used to identify the presence of clonally dominant and subclonal mutations through deep sequencing of ctDNA. Thirty-three samples (27 tumour, six normal) were selected for Affymetrix SNP 6.0 genotyping arrays to determine genomic architecture and copy number alterations (CNAs), and the exomes of 25 samples (19 tumour, six normal; Table 1) were sequenced to a median exonic coverage of 70.7-fold across different samples (see supplementary material, Table S1 and Figure S1). The 19 tumour samples were used as index discovery samples to identify sets of mutations for interrogation by deep amplicon sequencing 28 (median *>* ×5000 coverage) to: (a) confirm predicted mutations; (b) determine the presence/absence of mutations in specific samples; and (c) infer clonal diversity estimates both within and between samples from the same patient. For 29 tumour samples, RNA was extracted for gene expression profiling by Affymetrix U133 2.0 arrays.

**Table 1:** Case descriptions for the six HGSC patients profiled in this study

	Case no.	Age	Stage	Treatment	BRCA status	Progression-free survival (months)	Overall survival (months)	Patient status	Number of samples	Index samples (exome)	Index samples (Affy SNP 6.0)	Index samples (Affy U133 expression)
Spatial	1	56	IV	IV CP×6	Unknown (declined)	24	29	Alive, recurrent disease, 3^rd^ line chemo	**4** (right ovary, quadrants 1a–d)	**4** (All and normal)	**4** (All and normal)	**4** (right ovary, quadrants 1a–d)
	2	59	IIIA	IV/IP CP×3, IV×3	Screen negative	27	27	Alive, no evidence of disease	**4** (right ovary, quadrants 2a–d)	**4** (All and normal)	**4** (All and normal)	**4** (right ovary, quadrants 2a–d)
	3	64	IIIC	IV/IP CP×6	Unknown (moved)	9	9	Unknown (moved)	**3** (3a, right ovary; 3b, left ovary; 3c, posterior cul de sac)	**3** (All and normal)	**3** (All and normal)	**3** (3a, right ovary; 3b, left ovary; 3c, posterior cul de sac)
	4^*^	79	IIIC	IV CP×6	Screen negative	15	17	Alive, recurrent disease, 2^nd^ line chemo	**10** (4a–e, right ovary; 4f–i, left ovary; 4j, left fallopian tube)	**1** (4a and normal)	**9** (4a–i, and normal)	**9** (4a–e, right ovary; 4f–i, left ovary)
	5^*^	73	IIIC	IV CP×6	Unknown (declined)	17	17	Alive, no evidence of disease	**8** (5a, right ovary; 5b–e, left ovary; 5f, left iliac node; 5g, left para-aortic node; 5h, left fallopian tube)	**5** (5a–c, f, g and normal)	**5** (5a–c, f, g and normal)	**7** (5a, right ovary; 5b–e, left ovary; 5f, left iliac node; 5g, left para-aortic node)
Temporal	6	65	IIIC	Primary Rx: IV CP×6 Recurrence Rx; 21 cycles (multiple agents)	Screen negative	13	74	Dead of disease	**2** (6a, omentum (primary surgery); 6b, left ovary (recurrence))	**2** (both and normal)	**2** (both and normal)	**2** (6a, omentum (primary surgery); 6b, left ovary (recurrence))

Additional details of all methods are described in the Appendix (see supplementary material).

## Results

### Intratumoural mutational diversity in HGSCs

The somatic mutational profiles of synchronous spatially separated samples (removed during primary surgical staging) were compared in cases 1–5 (see supplementary material, Figures S2–S6). In case 6, we compared a primary sample to a recurrence obtained 42 months later, following multiple chemotherapeutic regimens (total 21 cycles) (see supplementary material, Figure S7). A total of 1349 somatic mutations in all samples were confirmed with deep amplicon resequencing (median 5079-fold coverage; see supplementary material, Table S2), of which 404 were unique mutations (334 non-synonymous, 46 synonymous and 24 small indels), ranging from 31 to 137 unique mutations per case. A panel of shared, identical mutations, indicative of a single ancestral clonal origin, was evident, with 55, 30, 16, 15, 19 and 32 mutations in cases 1–6, respectively. All cases except case 1 harboured a mutation in *TP53*, with the identical mutation present in all samples from that individual (see supplementary material, Table S2), supporting a monoclonal origin with a *TP53* mutation in the ancestral clone.

**Figure 1:**
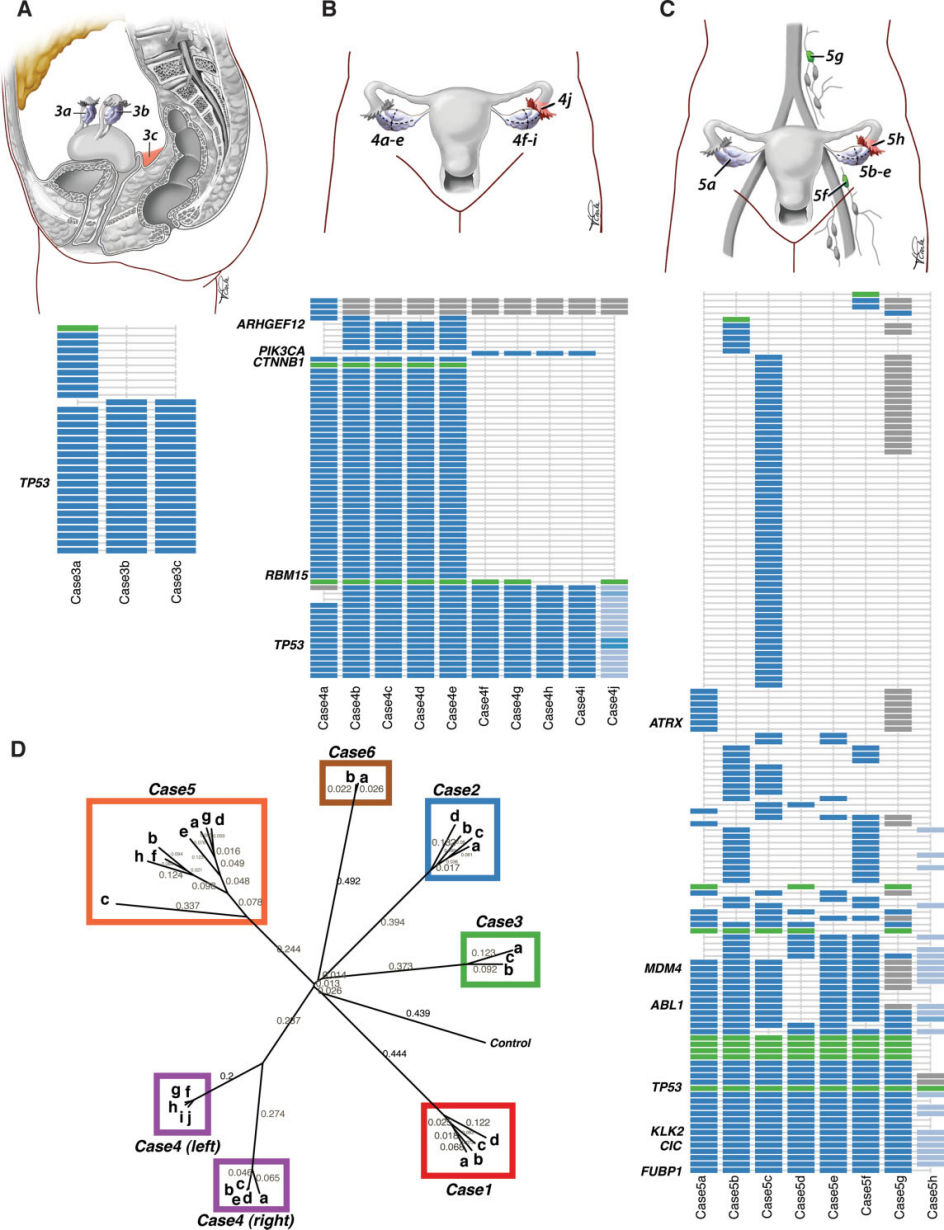
Intratumoural mutational profiles of HGS ovarian cancer. Anatomical sites and intratumoural mutational profile for cases 3 (A), 4 (B) and 5 (C); point mutations are shown in blue, indels in green; grey indicates a predicted mutation where validation by deep sequencing was inconclusive; light blue indicates allelic frequencies (counts of non-reference allele/total depth of coverage) in the fallopian tube lesion. (D) Phylogenetic tree of mutational profiles of cases 1–6, depicting evolutionary branching patterns reflective of clonal relationships between samples. The tree was computed using distance matrices based on Pearson correlation coefficients, followed by neighbour-joining cluster analysis. The control sample represents the ‘root’ whereby data were generated with no aberrations. Neighbour-joining distances are shown along the branches of the tree, which reflect genetic distances between branching points; longer branches indicate more genomic differences.

Patterns of mutation conservation between samples from the same patient varied widely across the six cases (51.5 *±* 30.7% mutations present within all samples of a case (Figure 1A–C; see also supplementary material, Table S3)). Case 5 had the lowest conservation of mutations (10.2%; Figure 1C), whereas case 6 had the highest (91.4%). Examination of mutations shared in samples of a single tumour mass, ie case 1a–d (right ovary), case 2a–d (right ovary), case 4a–e (right ovary), case 4f–i (left ovary) and case 5b–e (left ovary), had an average of 63.0 *±* 29% mutations present in all samples. Case 4f–i had the highest conservation of mutations (94.4%), followed by case 4a–e (81.2%), case 1a–d (67.1%), case 2a–d (55.6%) and case 5b–e (17.6%) (see supplementary material, Table S3). Non-synonymous mutations in known cancer genes not present in all samples included *PDGFRB* and *SH3GL1* in case 2 (see supplementary material, Figure S3) and *PIK3CA*, *CTNNB1* and *RBM15* in case 4 (Figure 1B). Case 5c harboured 54 (of 102 total) mutations that were not found in the other left ovary samples, which contained 66, 33 and 45 total mutations, respectively. Phylogenetic analysis (Figure 1D) revealed a common clonal ancestry of all samples in case 5, but with an ‘early’ divergence of 5c.

Due to highly disparate mutational profiles in left and right samples for case 4, we correlated the findings with histopathological review. The right ovary from case 4 (diagnosed and treated as HGSC) exhibited mixed histology of predominately high-grade endometrioid pattern, with gland formation and a minor HGSC component, while the left ovary, left fallopian tube and all metastatic sites consisted exclusively of HGSC (see supplementary material, Figure S8). Although the right ovarian tumour was p53 IHC-positive regardless of subtype, the expression of WT1 was negative in the endometrioid regions and positive in HGSC areas, as expected 34. All ten tumour samples (including left fallopian tube lesion case 4j) shared the identical mutation in *TP53* (chr17, 7518290, p.239N *>* S), along with 16 other mutations (Figure 1B), indicating a common cell of origin despite completely distinct histological profiles (see supplementary material, Figure S8). Forty-three mutations present in the right ovary (mixed histology) were not present in the samples from the left (all HGSCs; Figure 1B). As the index exome for this case was restricted to the right set of samples, we subjected all samples to the PGM Ion AmpliSeq Cancer Panel. We detected a *PIK3CA p*.546Q *>* R hotspot mutation in case 4f–i (left ovary) that was absent from case 4a–e. Consistent with endometrioid histology, we also detected a *CTNNB1* mutation in case 4a–e that was absent from case 4f–i 35.

Collectively, these results reflect extensively divergent mutational profiles in spatially separated samples, with significant evolutionary selection due to regional environments and highly unique evolutionary trajectories.

### Mutational events in the ancestral clone are detectable in cell-free circulating tumour DNA from plasma

**Figure 2:**
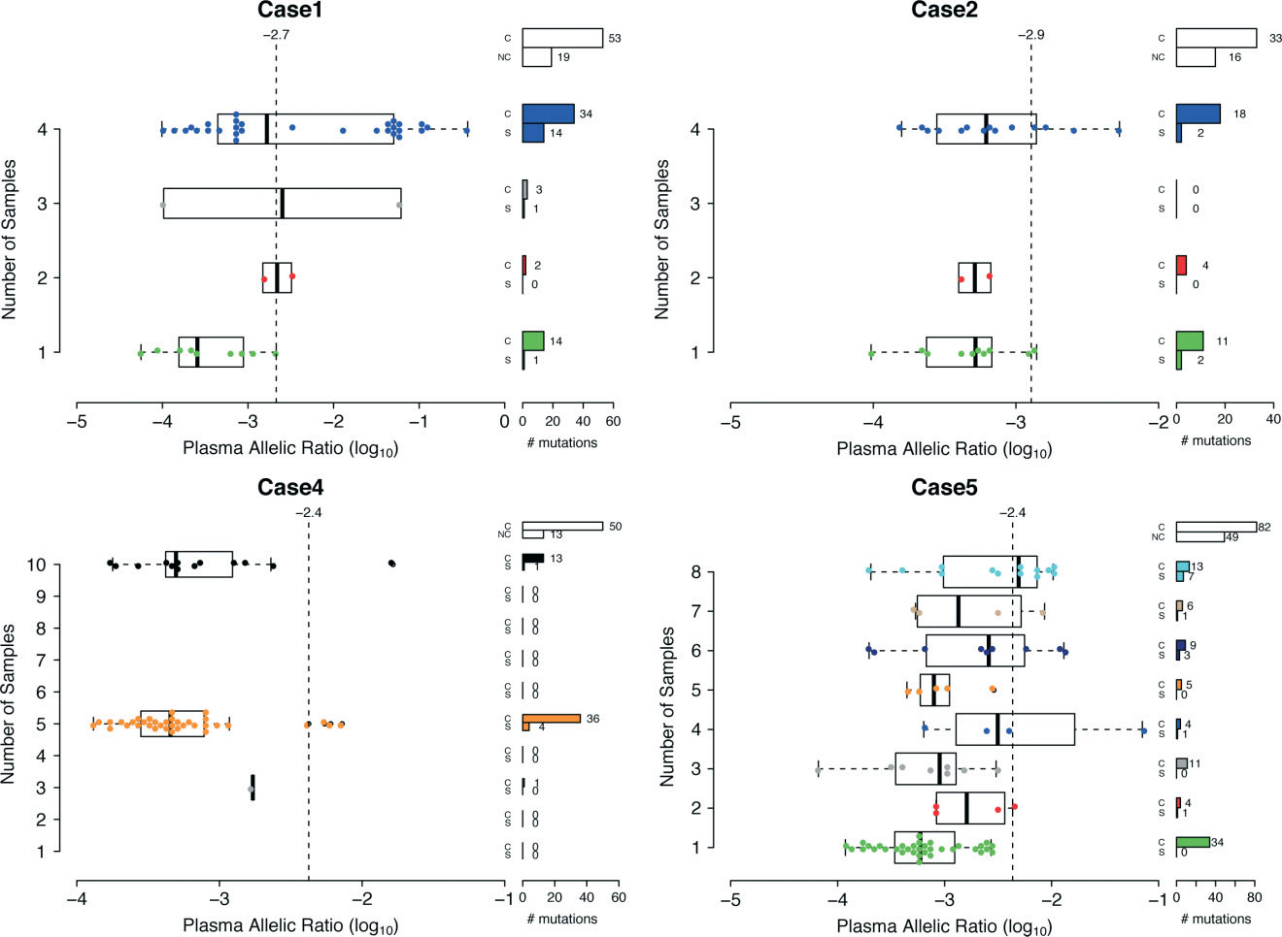
Deep sequencing results of cell-free circulating tumour DNA from the plasma in cases 1, 2, 4 and 5. The distribution of plasma variant allelic ratio (log10 scale) is separated by the number of tumour samples in which the mutation was originally discovered. Cases 4 and 5 both also include the fallopian tube when counting the number of tumour samples. The number of mutations based on positions with coverage (c), no coverage (nc) and those with coverage that were significantly (s) detected, based on the binomial exact test (adjusted *p <* 0.05), are shown. The minimum variant allelic ratio detectable using the exact test for each patient is denoted by the vertical dashed line.

Plasma samples collected pre-operatively and pre-anaesthesia were available for four cases from which ctDNA was extracted and subjected to targeted deep-sequencing of validated somatic mutations (see supplementary material, Appendix). We assayed 72 mutations in case 1, 49 in case 2, 63 in case 4 and 131 in case 5. Across the four cases, 215/312 (69%) mutations contained sequencing coverage and 38/215 (18%) were detectable with mutation allele frequency above statistical background noise (binomial exact test, false discovery rate *<* 0.05) (see supplementary material, Table S2). Cases 1 and 5 were enriched for the presence of mutations in the plasma that were also present in all intrapatient tumour samples, and thus part of the ancestral clone (Fisher exact test, *p <* 0.05; Figure 2). Among the mutations conserved in all primary tumour samples and detected in the plasma were the *TP53* mutation (chr17, 7518290 T *>* C) in case 4, 12 mutations in case 1, two mutations in case 2 and seven mutations in case 5. Thus, in each patient at least one mutation representative of the ancestral clone was detected. Fourteen mutations (two in case 1, two in case 2, four in case 4 and six in case 5) were also detected in ctDNA, although they were only present in a subset of the primary tumour samples of a given patient, indicating that subclonal mutations might also be amenable to monitoring in ctDNA.

We noted that the proportion of mutations detected in ctDNA was non-uniformly distributed across the four cases. In particular, cases 1 and 5 exhibited higher sensitivity (16/53 and 13/82 mutations, respectively) than cases 2 and 4, suggestive of heterogeneous rates of shedding tumour DNA into the circulation.

### The intratumoural variation in genomic architectures of HGSCs

**Figure 3:**
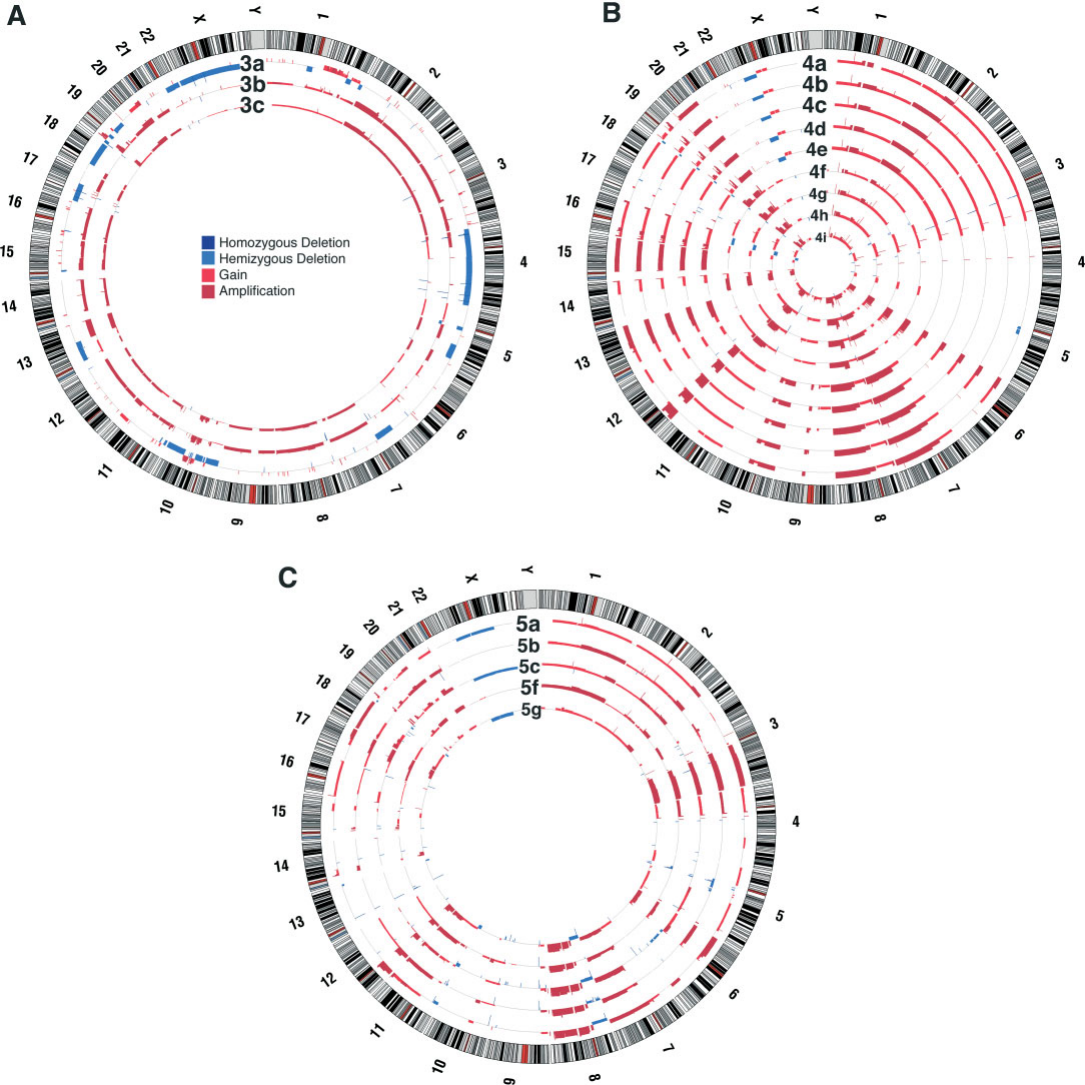
Intratumoural genomic architecture profiles of HGS ovarian cancer. Genomic copy number architecture of intrapatient samples using Circos; samples are arrayed in concentric circles as whole-genome profiles for cases 3 (A), 4 (B) and 5 (C). Colours represent the various copy number states: dark blue, segmental homozygous deletions; blue, hemizygous deletions; red, segmental gains; dark red, amplifications. Amplitude of each segment on the track represents the logR value of the segmental copy number change.

**Figure 4:**
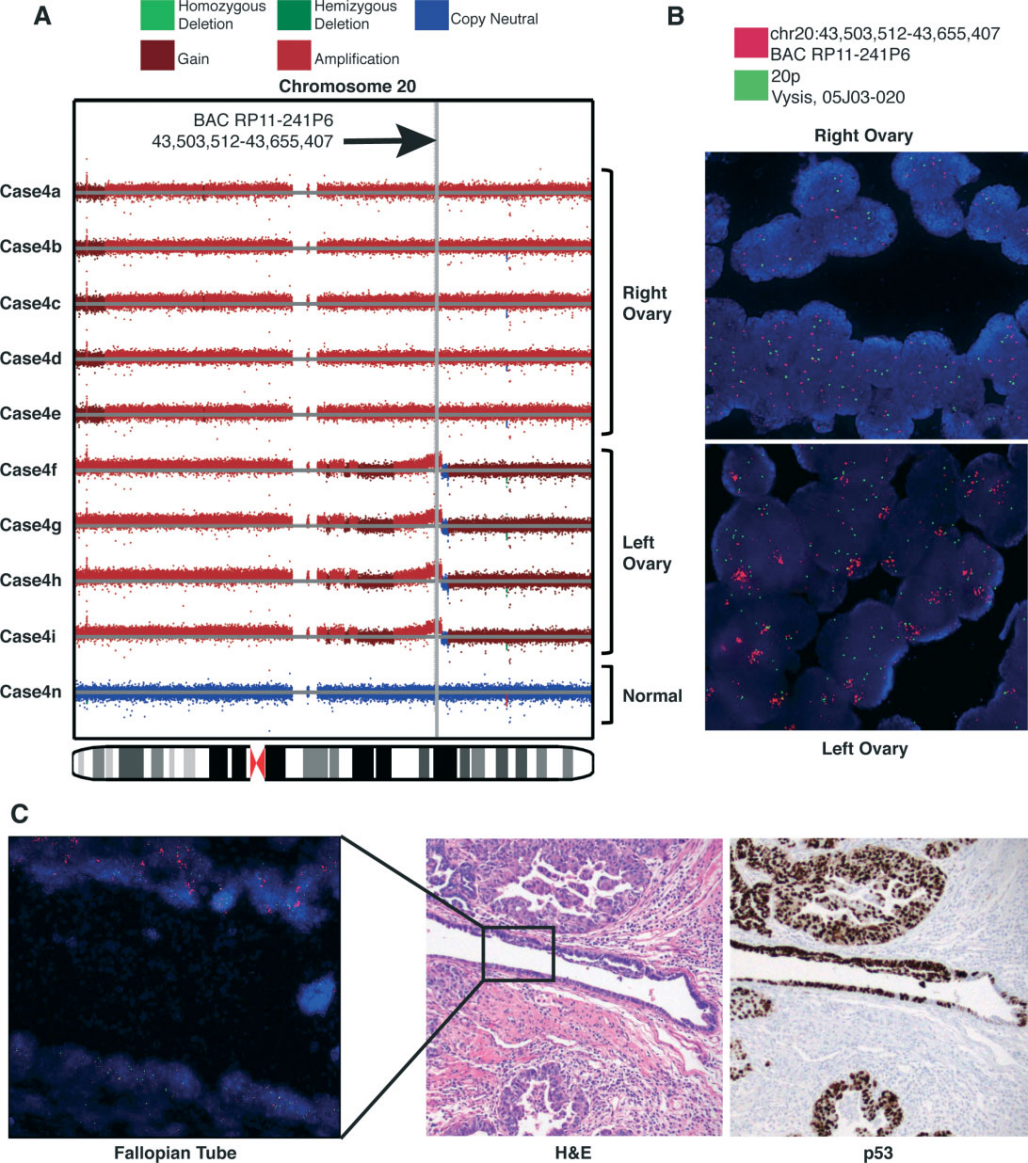
(A) Copy number alteration (CNA) and (B) fluorescence *in situ* hybridization (FISH) comparisons between right (a–e) and left (f–i) ovaries of case 4 at chromosome 20. The 20p control probe is labelled in spectrum green (Vysis, cat. no. 30–2520200), and Region 2 using BAC RP11-241P6 is labelled with spectrum orange (Vysis, Nick Translation Kit, cat. no. 32–801300). Right ovary (a–e) shows aneuploidic gain; left ovary (f–i) shows amplification of Region 2. (C) Both populations of cells carrying the chr20 CNA are found in the molecularly fixed, paraffin-embedded, early tubal high-grade serous carcinoma of the left fallopian tube (FT). FT FISH image corresponds to box inset in the H&E serial section shown at ×20 magnification. p53 immunopositivity highlights FT lesion in serial section, consistent with the presence of the same TP53 missense mutation (g.chr17, 7577565 T *>* C; c.716A *>* G; p.N239S) in all case 4 samples. Magnifications = (IHC image) ×20; (FISH images) ×63.

Copy number analysis from high-density genotyping arrays revealed highly disrupted karyotypes in all samples, and heterogeneous variation of genomic architectures in samples from different patients (intertumoural heterogeneity) (see supplementary material, Table S4). While the overall copy number landscapes between samples from cases 1, 2 and 6 were highly similar (see supplementary material, Figure S9), we observed substantial intratumoural heterogeneity between samples from cases 3–5 (Figure 3A–C). We first focused on extreme CNAs by examining genes affected by high-level amplifications and homozygous deletions between samples of the same patient (see supplementary material, Table S5). The most extreme example of copy number diversity was found between samples from the left and right ovaries of case 4, consistent with the mutation profiles. Across the samples in case 4, 631 genes were altered by homozygous deletions and 852 genes were altered by high-level amplifications. None of these alterations was observed in all nine tumour samples. The samples from cases 4a–e and 4f–j form distinct branches of the phylogeny and are at least as divergent from each other as from other patient samples. We validated three segmental amplifications in case 4 in one representative sample from the left and the right ovary, using fluorescence *in situ* hybridization (FISH): a 150 kb region, chr20, 43,503,512–43,655,407, predicted to have a high-level amplification in the left ovary of case 4 with only low-level amplification in the right ovary (Figure 4); an amplified region, chr6, 47,952,581–48,122,073 (see supplementary material, Figure S10A) and a highly amplified region, chr12, 25,863,200–26,026,351 region (see supplementary material, Figure S10B).

Several genes in the Cancer Gene Census 36 were altered by extreme CNA events in sample subsets (see supplementary material, Table S5). Case 1a–c (but not case 1d) exhibited homozygous deletion of tumour suppressor *NF1* in an approximately 190 kb region on chr17, 26,496,299–26,686,045, harbouring *NF1*, *OMG*, *EVI2B* and *EVI2A* (see supplementary material, Figure S11A). FISH assays probing this event revealed subpopulations of cells containing homozygous deletion of *NF1* and all cells containing monosomy of chr17, confirming that homozygous deletion of *NF1* was not in the ancestral clone. The same scenario was also observed in case 3, which harboured *NF1* homozygous deletions in only two of the three samples (see supplementary material, Figure S11B). Observations of subclonal *NF1* deletions reinforced evidence from the mutation data that important driver mutations may be acquired late in the evolutionary history of the tumour.

**Figure 5:**
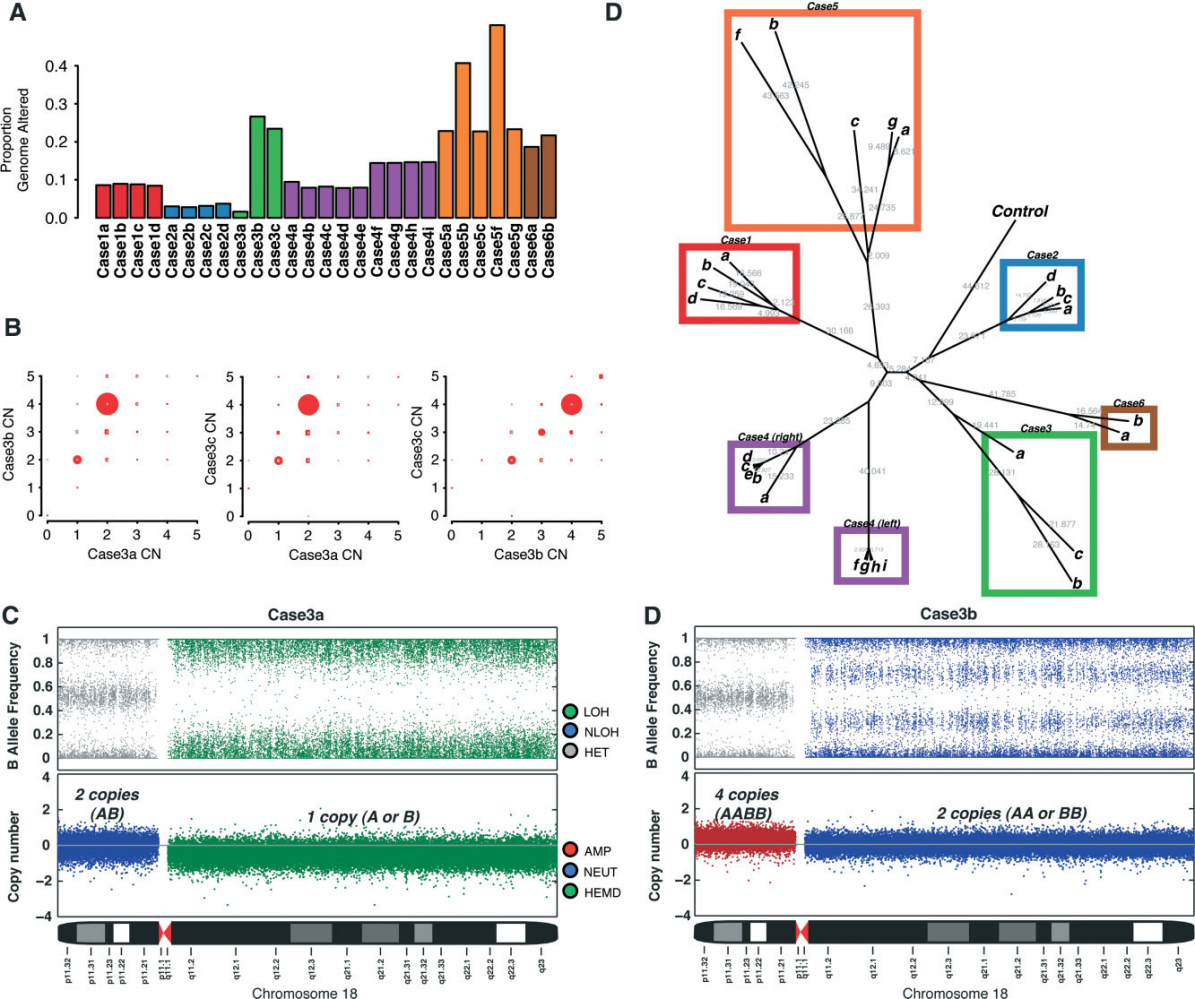
Evolutionary sequential compound copy number analysis. (A) Analysis of proportion of the genome that was altered by sequential compound events. Compound events include copy neutral LOH (NLOH) and amplified LOH (ALOH) regions, which indicates the occurrence of more than one copy number event in sequence (eg deletion followed by amplification of remaining allele results in ALOH). (B) Pairwise comparison of copy number samples within case 3. The number of genes with a specific predicted discrete copy number (CN) is represented by the size of the dot. Genes that also have the same zygosity (LOH or heterozygous) status between the two samples are coloured red; otherwise they are grey. (C) Doubling of chromosome 18 in case 3b relative to case 3a. Deletion (green) in 18q in 3a is observed as NLOH (blue) in 3b; amplification of 18p in 3b is balanced, indicating doubling of both diploid alleles in 3a. ‘A’ and ‘B’ genotypes are used to denote the two alleles. (D) Phylogenetic tree of discrete compound events. Genes were assigned an integer value representing the weight of observing compound events: 2, ALOH; 2, NLOH; 2, homozygous deletion; 1, hemizygous deletion; 0, diploid heterozygous; 0, allele-specific amplification. Euclidean distance was computed between pairs of tumour samples and a control (which consists of zeros for all genes) and neighbour-joining cluster analysis was used to generate the tree.

In addition to high-level amplifications and homozygous deletions, we compared the overall genome architecture based on loss of heterozygosity (LOH) profiles inferred from allelic intensity ratios (see supplementary material, Appendix). All intrapatient and intratumour samples in the six cases harboured LOH in chromosome 17 (see supplementary material, Table S4), supporting evidence that this is among the earliest aberrations in the tumourigenic process. To investigate events that may further indicate continual evolution of the genomic architecture, we identified chromosomal aberrations, such as copy-neutral LOH (NLOH) and amplified LOH (ALOH), that arose from at least two sequential (compound) genomic modifications. Despite the similarities of overall proportion of the genome altered by copy number events between intrapatient samples (Figure 3A–C; see also supplementary material, Figure S9), the relative proportion of the genome altered by compound copy number events varied between samples of cases 3 and 5 (Figure 5A). For cases 3b and 3c (but not 3a) we found evidence of whole-genome duplication events (Figure 5B). Segmental deletions in case 3a which were observed as NLOH events (doubling of the remaining allele) in cases 3b and 3c and diploid heterozygous regions in case 3a were observed to be doubled to four balanced copies in cases 3b and 3c (Figure 5C; see also supplementary material, Figure S12A). Overall, 17 chromosomes in cases 3b and 3c showed evidence of doubling. The remaining chromosomes (4, 8, 11, 13 and 19) appeared to have undergone concurrent segmental aneuploid events to both case 3a and 3b preceding or following genome doubling (see supplementary material, Figure S12B, C), suggesting continual accrual of genomic aberrations after clonal divergence of case 3a from the ancestral clone (Figure 5D). In case 5, samples 5b and 5f exhibited a higher proportion of the genome harbouring compound aberrations (mean 0.43) than the other samples–5a, 5c and 5g (mean 0.23) (Figure 5A). Case 5c harboured the highest number of mutations, with 54 of 102 mutations unique to that sample. As such, the evolution of genome architecture for case 5 appears to be independent of evolution at nucleotide scales. In case 3, substantial intratumoural variation in segmental copy number alteration profiles accrued with relatively conserved single nucleotide patterns. These results suggest that evolutionary trajectories of copy number and mutational profiles can vary independently, and must both be considered to form a complete picture of clonal divergence.

### The origins of fallopian tube lesions and reconstruction of HGSC evolutionary histories

For two cases (4j and 5h) we profiled fallopian tube lesions obtained at primary surgical staging. The lesion in case 4j was a small focus of invasive carcinoma with involvement of the fallopian tube mucosa, while 5h showed invasive carcinoma within the fallopian tube wall, without mucosal involvement. It is believed that HGSC may originate from the fimbriated end of the fallopian tubes and shed cells into the peritoneal cavity, spreading transcoelomically. We deep-sequenced the set of somatic mutations over all primary tumour samples for cases 4 and 5 in the FT lesions to examine their evolutionary histories. In case 4j we identified 17 mutations (26.2% of all mutations in case 4; Figure 1B), and in case 5h we found 44 mutations (32.1% of all mutations in case 5; Figure 1C). In both cases the somatic mutations in *TP53* were among the set of mutations detected in the FT.

The FT lesion in case 5 harboured seven mutations that were not present in all samples, indicating that the clones comprising case 5h were unlikely to have been ancestral to the tumours. Rather, phylogenetic analysis suggested that case 5h had closer clonal relationships with case 5b and 5f (left ovary and para-aortic lymph node, respectively) compared to cases 5a, 5d, 5e and 5g (Figure 1D). We suggest that case 5h is therefore more likely to be a metastatic implant in the FT rather than a precursor or early lesion. By contrast, case 4j shared all mutations present in samples taken from the left ovary with the exception of *PIK3CA p*.546Q *>* R, found at a low frequency (3% of the total 4671 reads) and only present in the region of the lesion closest to the ovary. Conversely, the fallopian tube lesion did not contain the mutation in *CTNNB1* present in the right ovary samples, suggesting that it was acquired after the 17 mutations and was restricted to the endometrioid component of the right tumour.

Results from *PIK3CA* mutation sequencing suggested the FT lesion in case 4 was composed of heterogeneous subclones. FISH on three amplified regions of chromosomes 6, 12 and 20 (predicted in the copy number data; see above) which were specific to the right (chr12) or left (chr6, chr20) samples, revealed two coexisting populations of nuclei in the FT lesion–those that had high-level amplification on chr20 (BAC RP11-241P6) similar to the left ovary, and aneuploidic gain of the region as seen in the right ovary (Figure 4). The region profiled on chromosome 6 in the FT showed copy number patterns similar to the right ovary (see supplementary material, Figure S10A), while the chr12 region in the FT resembled the left ovary (see supplementary material, Figure S10B). Taking mutational and copy number data together, case 4 follows a monoclonal hierarchy in which two dominant clones emerged in the FT and gave rise to the divergent, histologically distinct populations in the right and left ovaries. To our knowledge, this is the first example of two distinct but related precursor clonal populations in the fallopian tube *in situ* lesion, with molecular data indicating that divergent cell populations in tubal mucosal lesions can evolve before extratubal spread of disease.

### Intra-sample clonal diversity revealed by cellular frequency analysis of mutational profiles

**Figure 6:**
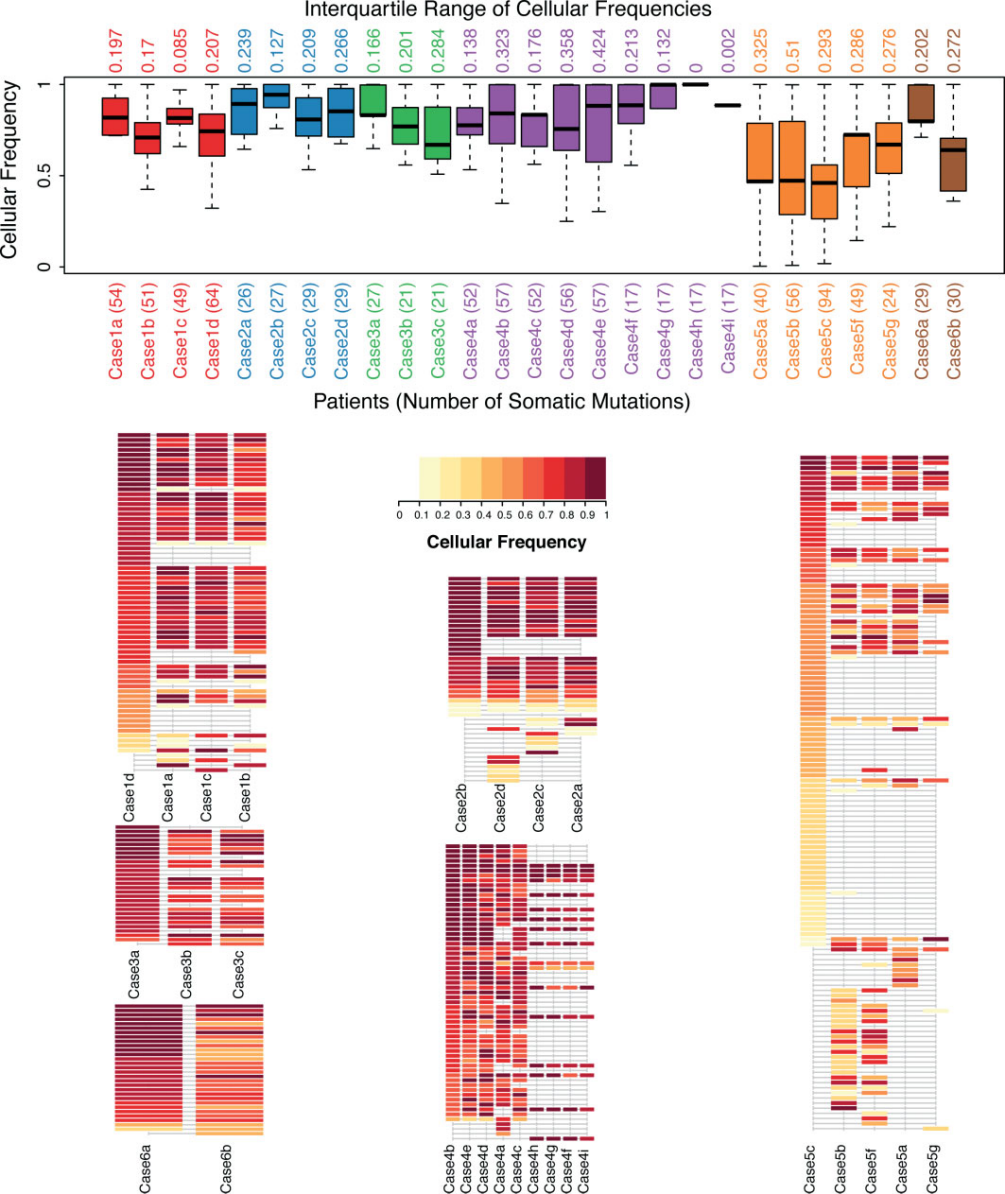
Intra-sample clonal diversity spectrum of HGSCs. (A) Distribution of cellular frequency estimates over mutations in each sample (estimated using PyClone), indicating statistically significant variation both within and between samples of the same case. (B–G) Profile of cellular frequencies for all cases, where darker shades of red indicate increasing cellular frequency estimates.

We integrated genomic aberrations at all scales, including mutations, copy number and LOH, to infer the degree of clonal diversity existing *within* each sample. Using a Dirichlet process statistical model, PyClone 28 (see supplementary material, Appendix and Figure S13), we inferred the clonal population structure of each sample. The output of the PyClone model is an estimate of the proportion of cells in the sample harbouring each mutation in the input (cellular frequency). Each sample from cases 1–6 exhibited variance in the distribution of cellular frequencies (Figure 6), suggesting presence of intra-sample clonal diversity. Case 5 showed the highest degree of intra-sample variation with the minimum interquartile range (IQR) of 0.276 (case 5g) and the maximum of 0.510 (case 5b). Case 1 showed the lowest degree of intra-sample variation, with 0.085, 0.170, 0.197 and 0.207 IQR in samples 1c, 1b, 1a and 1d, respectively. Intriguingly, two of the case 4 samples (4h and 4i) showed comparatively tight distributions, with IQRs of 0 and 0.002, respectively. Finally, case 6b (postchemotherapy sample) showed a higher degree of variation in clonal frequency than the primary sample, suggesting that clones present in equal proportions in case 6a emerged in different proportions after chemotherapy in case 6b.

### Diverse genomic landscapes associate with heterogeneous transcriptional profiles

**Figure 7:**
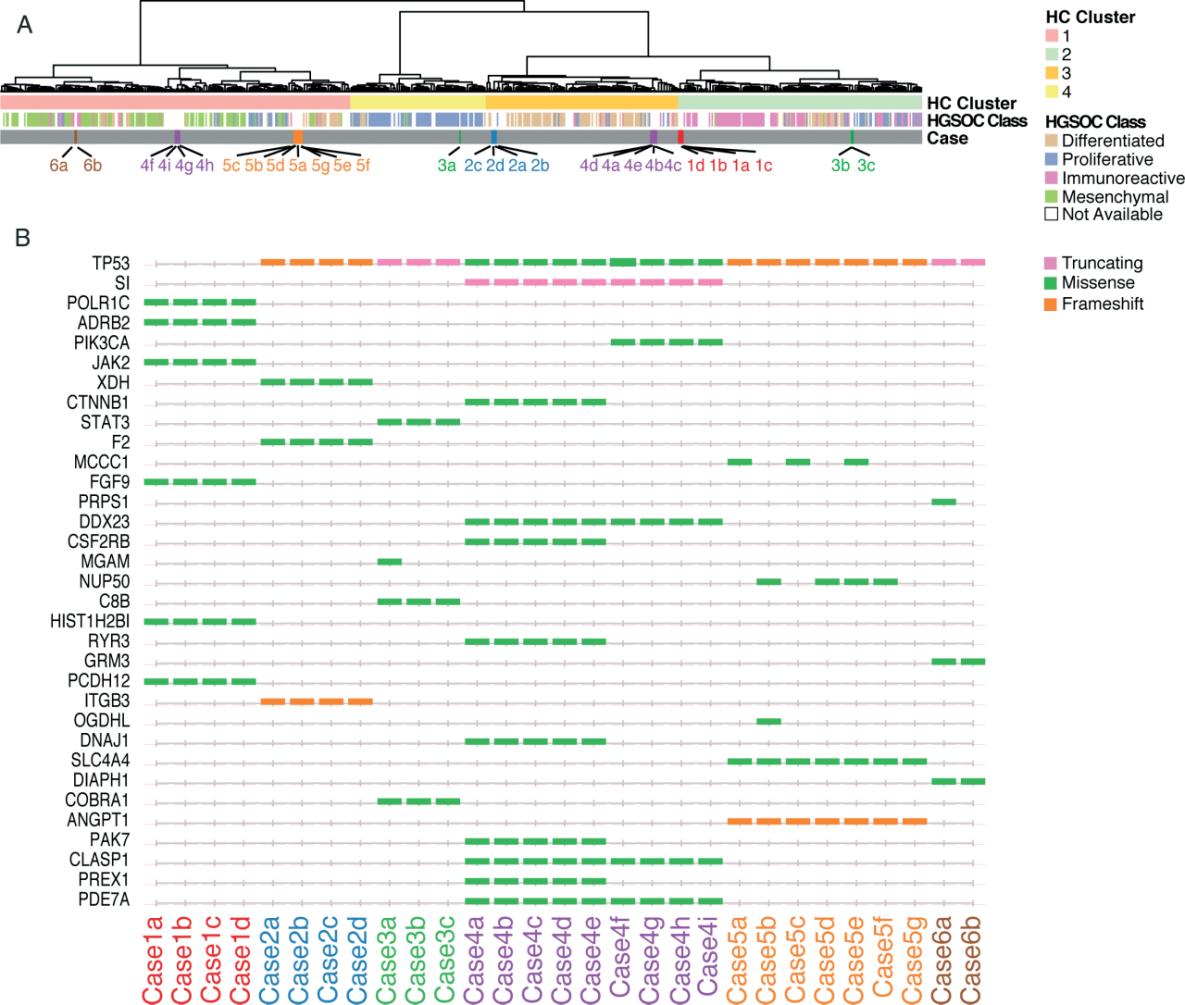
(A) Hierarchical clustering of the expression data in a cohort consisting of 594 TCGA HGSC samples and 29 HGSC samples, representing six cases analysed in this paper. The bars show four patient groups according to the hierarchical clustering of the samples (top bar), patient labels according to Tothill *et al* 37 classification (middle bar), and distribution of our samples across all 623 samples. Clusters C1, 2, 4, 5 from Tothill *et al* correspond to differentiated, mesenchymal, immunoreactive and proliferative labels shown in the figure. (B) Simultaneous analysis of mutations and expression profiles by DriverNet, nominating the mutations that had significant impact on expression networks.

We next analysed mRNA gene expression profiles to examine the extent of intratumoural transcriptional diversity. We clustered our 29 samples with the TCGA cohort of 594 HGSC samples downloaded from the TCGA portal (see supplementary material, Appendix) to determine how our samples were distributed in the TCGA population (Figure 7A). Consistent with previous reports 3–37, there were four clusters of patients representing HGSC subgroups, which we mapped to the TCGA groupings: differentiated, proliferative, immunoreactive and mesenchymal (Figure 7A). Samples from cases 1, 2, 5 and 6 clustered together such that expression profiles from within one patient were found to be adjacent in the dendrogram. By contrast, cases 3 and 4 exhibited widely discrepant expression profiles between samples from the same patient. The expression profile for case 3a clustered with the proliferative group, whereas cases 3b and 3c clustered with the immunoreactive group. Thus, 3a, 3b and 3c were placed in different major branches of the dendrogram (Figure 7A), consistent with divergent copy number profiles (Figure 5). In case 4, the left ovary samples (4f–i) clustered together within the mesenchymal group, while the right ovary samples (4a–e) were clustered together with the differentiated group. These results suggest that genomic diversity observed in these two patients may elicit changes in the transcriptional programme, reflective of phenotypic change.

### Genomic aberrations impacting transcriptional networks

Finally, we integrated gene expression and genomic mutation data to identify specific mutations impacting mRNA expression networks, using the DriverNet 38 algorithm (see supplementary material, Appendix). In total, DriverNet predicted 33 genes to be significantly associated with disrupted transcriptional networks (Figure 7B; see also supplementary material, Table S6), with *TP53* as the top-ranked gene.

In case 1, mutations in *SI,* *POLR1C,* *JAK2,* *FGF9,* *HIST1H2BI* and *PCDH12* were predicted to affect transcriptional networks (DriverNet; *p <* 0.05). *SI* and *POLR1C* mutations associated with disrupted expression of metabolic, TNF*α* and NF-*κ*B pathways, while *JAK2* associated with disrupted expression in adipocytokine, chemokine and Jak–STAT signalling, IFN*γ*, Notch and Sphingosine 1-phosphate (S1P) pathway genes. *FGF9* and *PCDH12* mutations were associated with altered expression in *FGF*, *MAPK* and *Wnt* signalling pathway genes (see supplementary material, Table S6). These mutations are strong candidates for tumourigenic drivers in case 1, as all six were present in all samples in addition to impacting expression. Other than *TP53*, genes with mutations in all samples predicted to impact expression were *XDH*, *F2* and *ITGB3* (case 2); *STAT3*, *C8B* and *COBRA1* (case 3); *PDE7A*, *CLASP1*, *DDX23* and *SI* (case 4); *ANGPT1* and *SLC4A4* (case 5), and *DIAPH1* and *GRM3* (case 6). DriverNet predictions consisting of mutations present only in a subset of samples of a patient included *MGAM* (case 3), *PIK3CA*, *CTNNB1*, *CSF2RB*, *RYR3*, *DNAJ1*, *PAK7* and *PREX1* (case 4), *MCCC1*, *NUP50* and *OGDHL* (case 5) and *PRPS1* (case 6).

These results illuminate a small subset of mutations potentially acting with *TP53* in the ancestral clone (present in all samples) that alter the transcriptional programme. By contrast, mutations associated with alteration in expression of transcriptional networks present only in a subset of samples represent candidate mutations for non-tumourigenic driver mutations acquired after divergence from the ancestral clone. As such, these represent mutations potentially altering the phenotypes of only a subset of primary tumour cells.

## Discussion

We have shown the range of genomic diversity at nucleotide, copy number and gene expression scales present in six patients with HGSC. These samples were resected prior to treatment, revealing intratumoural diversity in the tumours’ natural evolutionary state. Our results were consistent with recent reports 39 that considerable genomic diversity is measurable in HGSCs at the level of copy number. However, in some cases (cases 3 and 5) we observed large-scale copy number differences in spatially separated samples that were not evident at the level of mutational profiling, indicative of different mutational mechanisms operating independently in different parts of a tumour. Together, copy number and mutational profiling identified well-characterized mutations in genes such as *PIK3CA*, *CTNNB1* and *PDGFRB*, as well as homozygous deletions in *NF1*, that were not present in all samples of the same case. With the exception of *TP53*, our data suggest that well-known, actionable driver mutations may only be partially represented if only a single sample is considered per patient. Our results establish that embedded within the context of extreme intra- and intertumour heterogeneity, *TP53* mutation remains the most stable genomic feature in HGSC. However, as efforts towards precision medicine informed by mutational landscapes of tumours reach maturity, it will be imprudent to ignore the degree to which regional genomic variation is present in primary samples prior to any treatment-related selective pressures. Moreover, studies of evolution in cancer through analysis of serial biopsies (eg through pre- and post-treatment samples, or comparisons of primary and metastatic samples), will need to account for evidence of divergent profiles simply due to regional sampling bias.

Mutational profiling revealed for the first time that mutations beyond *TP53* are present in FT lesions. In case 4, we used the full spectrum of mutations to establish that evolutionary trajectories of two histologically and karyotypically distinct tumours within a single patient arose from a common aetiology, with 26.2% of all mutations (including *TP53*) conserved in the tubal lesion. Our results indicate how histologically distinct cell populations in the same patient can be linked by common aetiology in the FT. In contrast to HGSC, endometrioid tumours are thought to develop from atypical ovarian endometriosis, with the FT merely acting as a conduit for endometrial epithelium to spread to the ovary (reviewed in 40). The possibility of the FT playing a causative role in rare cases of endometrioid carcinoma, or mixed endometrioid–serous carcinoma, has not yet been explored. Mutations in case 5 revealed the FT lesion to be a metastatic implant more closely related to a lymph node metastasis than to the ovarian samples. Thus, future investigations into aetiological underpinnings of HGSC will likely benefit from mutational comparison to extra-tubal sites to definitively establish the evolutionary origin of FT lesions.

We noted an extreme case of unexpected genomic conservation. In case 6, samples were obtained at primary surgery and after 42 months, with 21 cycles of multi-agent chemotherapy in between. The two samples exhibited near-identical genomic landscapes. Coupled with the long survivorship of this patient, this observation represents an intriguing anecdote. Larger studies will be needed to establish if stable clonal population structure is a feature of long survivorship.

Finally, as proof of concept, examination of plasma ctDNA suggested that representative mutations in the ancestral clone and mutations present in subclones could be detected in ctDNA, although sensitivity to detect mutations varied from patient to patient. This implies that clinical sensitivity analyses will need to be undertaken to establish the generalizability of ctDNA analysis to clonally diverse tumours.

In summary, our study unveils the extensive genomic diversity in primary, untreated, high-grade serous cancers of the ovary prior to treatment-related selection pressures, and illuminates highly individualized evolutionary trajectories that will require detailed consideration if curative therapeutic strategies are to be achieved.

## Data and materials availability

All sequencing data will be deposited at the European Genome-Phenome Archive (EGA, http://www.ebi.ac.uk/ega/), which is hosted by the EBI, under accession number EGAS00001000547.

## Supplementary material on the internet

The following supplementary material may be found in the online version of this article:

Appendix: additional details of all methods and patient clinical histories

**Figure S1.** Distribution of sequence coverage of targeted exons for the 25 (19 tumour and six normal samples) exome capture libraries.

**Figure S2.** Intratumoural mutational profile for case 1.

**Figure S3.** Intratumoural mutational profile for case 2.

**Figure S4.** Intratumoural mutational profile for case 3.

**Figure S5.** Intratumural mutational profile for case 4.

**Figure S6.** Intratumoural mutational profile for case 5.

**Figure S7.** Intratumoural mutational profile for case 6.

**Figure S8.** Immunohistochemistry (IHC) profiles for case 4 indicate mixed histology.

**Figure S9.** Genomic copy number architecture of intra-patient samples using Circos.

**Figure S10.** Copy number alteration and fluorescence in situ hybridization comparisons between right and left ovaries and fallopian tube of case 4 at chromosomes 6 and 12.

**Figure S11.** Heterogeneous NF1 homozygous deletions in cases 1 and 3.

**Figure S12.** Comparison of cases 3a and 3b reveals genome doubling.

**Figure S13.** The pyClone model shown as a probabilistic graphical model.

**Table S1.** Median and standard deviation of coverage for the exons for the 25 (19 tumour, six normal samples) exome capture libraries.

**Table S2.** Omnibus table of validated somatic SNVs and indels.

**Table S3.** Mutation summary table.

**Table S4.** Copy number segments for intratumour samples of six cases.

**Table S5.** Extreme copy number segments for intratumour samples of six cases; homozygous deletions and high-level amplifications.

**Table S6.** Ranked list of candidate driver genes.
