# Supplementary material for: Distinct evolutionary trajectories of primary high-grade serous ovarian cancers revealed through spatial mutational profiling
Source: J Pathol. 2013 Aug 6;231(1):21–34. doi: 10.1002/path.4230 (PMC3864404; doi:10.1002/path.4230)
Supplement: Supplementary file 1 — Figure S1. Distribution of sequence coverage of targeted exons for the 25 (19 tumour and six normal samples) exome capture libraries. [file path0231-0021-sd1.pdf]

Distinct evolutionary trajectories of primary high-grade serous ovarian cancers revealed through spatial mutational profiling
(Supplementary Appendix)

Ali Bashashati, Ph.D.[1,*], Gavin Ha, B.Sc.[1,*], Alicia A Tone, Ph.D.[2,*], Jiarui Ding, M.Sc.[1,3], Leah M Prentice, Ph.D.[2], Andrew J L Roth, B.Sc.[1], Jamie B Rosner, B.Sc.[1], Karey A Shumansky, M.Sc.[1], Steve E Kalloger, M.Sc.[2], Janine Senz, B.Sc.[2], Winnie Yang, B.Sc.[2], Nataliya Melnyk, B.Sc.[2], Margaret T Y Luk, B.Sc.[4], Kane Tse, B.Sc.[5], Thomas Zeng, M.Sc.[5], Richard A Moore, Ph.D.[5], Yongjun Zhao, D.V.M.[5], C Blake Gilks, M.D.[6], Marco A Marra, Ph.D.[5], Stephen Yip, M.D. Ph.D.[7], David G Huntsman, M.D.[2,7], Jessica N McAlpine, M.D.[8], and Sohrab P Shah, Ph.D.[1,7]

[1]Department of Molecular Oncology, British Columbia Cancer Agency, Vancouver, Canada
[2]Centre for Translational and Applied Genomics, British Columbia Cancer Agency, Vancouver, Canada
[3]Department of Computer Science, University of British Columbia, Vancouver, Canada
[4]Department of Anatomical Pathology, Vancouver General Hospital, Vancouver, Canada
[5]Canada's Michael Smith Genome Sciences Centre, British Columbia Cancer Agency, Vancouver, Canada
[6]Genetic Pathology Evaluation Centre, Vancouver General Hospital, Vancouver, Canada
[7]Department of Pathology and Laboratory Medicine, University of British Columbia, Vancouver, Canada
[8]Department of Gynecology and Obstetrics, University of British Columbia, Vancouver, Canada

[*]equal contribution

Contents

1 Case selection and ethical consent 3

2 Clinical case histories 3

3 Specimen preservation and histologic evaluation 5

4 Fallopian tube identification, coring and extraction 5

5 Fluorescent In Situ Hybridization 6

6 Sequencing and mutation identification 6

7 Affymetrix SNP6 analyses 9

8 Clonal frequency analysis 10

9 Affymetrix HT-HG-U133A 2.0 11
9.1 Sample preparation 11
9.2 Data processing 11

10 Phylogenetic analysis **11**

1 Case selection and ethical consent

Ethical approval was obtained from the University of British Columbia (UBC) Ethics Board. Women undergoing debulking surgery (primary or recurrent) for carcinoma of ovarian/peritoneal/fallopian tube origin were approached for informed consent for the banking of tumor tissue. Cases of high-grade serous carcinoma where more than one sample were collected in different anatomic locations (e.g., different locations within the ovary, omentum) or where material was available over different time periods (e.g., at primary surgery and at recurrence) were chosen for this analysis. Clinicopathologic and outcome data was collected by chart review. Consistent with the practice at UBC and the British Columbia Cancer Agency all patients with high-grade serous cancer are referred to the hereditary cancer clinic and offered genetic testing for BRCA1 and BRCA2 mutations (McAlpine et al., 2012; Schrader et al., 2012) (http://www.bccancer.bc.ca/HPI/CancerManagementGuidelines/HereditaryCancerProgram/referralinformation/hboccriteria.htm).

2 Clinical case histories

Case 1

Case 1 is a 56 year old woman of Persian descent, with no personal nor family history suggestive of hereditary breast or ovarian cancer syndrome (HBOC). She developed symptoms of early satiety, abdominal distension and bloating and was found on examination to have a large (20 cm) adnexal mass and ascites. She underwent suboptimal debulking surgery that included total abdominal hysterectomy (TAH), bilateral salpingoophorectomy (BSO), omentectomy, lymph node sampling, and resection of an anterior abdominal wall umbilical nodule (additional pelvic nodes considered unresectable were left in situ). Final pathology revealed stage IV high grade serous (HGS) carcinoma, involving both ovaries, uterine serosa, peritoneal nodules (including umbilical nodule), and nodal tissue with positive ascites. She received six cycles of IV carboplatin and paclitaxel (CP). Her CA125 level normalized after one cycle (258kU/L pre-operatively), and imaging was within normal limits at completion of treatment (i.e. residual disease left at debulking surgery no longer seen). She recurred 12 months after diagnosis (7 months from completion of therapy) with CT scan evidence of disease in her liver, perisplenic tissues and left iliac nodes. She received 3 cycles of second line CP that showed evidence of progression and is now on third line gemcitabine. The patient was referred to the Hereditary Cancer Program (HCP) at the BCCA (histology-based referral, no family history suggestive of HBOC). She initially filled out paperwork but declined to come in for an appointment and serum testing. Germline BRCA1/2 mutation status unknown. Four samples were obtained from the right ovary (four quadrants) as well as a serum sample for germline testing.Four samples were obtained from the right ovary (four quadrants) as well as a serum for germline testing.

Case 2

Case 2 is a 59 year old woman of Persian descent who noticed increasing abdominal girth and discomfort prompting her to visit her physician. Physical examination and imaging (CT scan) identified a large pelvic mass and free fluid in the pelvis and abdomen suggestive of ovarian cancer. She underwent optimal debulking surgery which included TAH, BSO, omentectomy, and biopsies (miliary/2 mm bowel serosa deposits in abdomen and pelvis). Pathology revealed HGS cancer evident in both ovaries, bowel serosal deposits, and positive ascites (stage IIIA). An intraperitoneal port had been placed during surgery and she received three cycles of IP/IV combination CP then switched to IV only CP for her remaining three cycles secondary to toxicity and port malfunction. Her CA125 normalized after two cycles (2000 kU/L preoperatively) and no post-treatment imaging was obtained. After two years of follow-up and with no evidence of disease (NED) the patient was discharged to the care of her local family practitioner. Registry and follow-up with primary physician confirms she is alive and without evidence of disease at 27 months from diagnosis. She had no family history suggestive of HBOC but given her HGS histology she was referred to the HCP. She underwent BRCA1/2 germline mutation testing, and no mutations were detected. Four samples were obtained from the right ovary (four quadrants) as well as serum for germline testing.

Case 3

Case 3 is a 64 year old woman of British descent who presented to her physician complaining of a several month history of bloating. Family history was limited but revealed colon and bladder cancer, and no HBOC-related cancers. On exam she had a pelvic mass and free fluid. She underwent TAH, BSO, omentectomy and optimal debulking with HGS carcinoma found in/on both ovaries and fallopian tubes, omentum, posterior cul-de-sac (miliary studding of all peritoneal surfaces) and positive ascites (stage IIIC). She received IV/IP CP for 6 cycles without interruption. Her CA125 which had been elevated to 265kU/L preoperatively was within normal range after 1 cycle. She attended genetic counseling for her personal risk of BRCA1/2 mutation but opted to wait for serum testing until returning to England to live. She was last seen nine months post diagnosis and staging, at which time she was without evidence of disease. Her current disease status and BRCA testing results are unknown. At her surgery, a single sample was obtained from the right ovary, left ovary, posterior cul-de-sac and omentum with inadequate tumor cellularity of the omental sample prompting exclusion of this site from analysis (total of 3 intraperitoneal surgical samples and saliva for germline testing).

Case 4

Case 4 is a 79 year old woman of British descent who noted several months of worsening urinary incontinence, new onset of abdominal distention, and fatigue. Exam and imaging confirmed a large (15cm x 12 cm) complex mass. CA125 level exceeded 2000 kU/L prior to surgery. She underwent surgical staging with optimal debulking. Pathology revealed mixed HGS and endometrioid carcinoma of the right ovary, with exclusively HGS found within the left ovary and all other metastatic sites, including the left fallopian tube, omentum and all peritoneal biopsies obtained in the abdomen and pelvis (stage IIIC). She received IV CP for 6 cycles with normalization of her CA125 level after 3 cycles. Her CA125 level from 6 months post-chemotherapy (11 months post surgical staging/diagnosis) had risen to 73kU/L although no evidence of disease could be seen on imaging, nor was detected on physical exam. She was asymptomatic and therefore observed. Four months later (15 months from diagnosis) she developed mild symptoms and imaging confirmed multiple peritoneal implants and retroperitoneal adenopathy. She has commenced second line chemotherapy with CP. Hereditary cancer referral and testing revealed no germline mutations of BRCA1/2. At her surgical staging she had five samples collected from her right ovary, four samples collected from her left ovary, and preservation of a left fallopian tube lesion in molecular fixative (total 10 surgical samples of carcinoma in addition to serum for germline analysis).

Case 5

Case 5 is a 73 year old woman of German, non-Jewish heritage who noticed increasing abdominal girth and was worked up for a possible malignancy. Imaging revealed an enlarged pelvic mass and a markedly elevated CA125 level (8100kU/L). She underwent optimal debulking surgery including resection of enlarged pelvic and para-aortic lymph nodes, omentectomy, TAH, and BSO. HGS cancer was identified in both ovaries and fallopian tubes, the left internal iliac, left and right para-aortic nodes, and washings (stage IIIC). Her omentum was negative for disease. She received standard IV CP for six cycles and at 17 months from diagnosis is without evidence of disease. She was counseled regarding testing for BRCA1/2 mutations but has not pursued genetic testing at present. At staging, a single sample was obtained from her right ovary, four samples obtained from her left ovary, one sample taken from a left iliac node, one from a left para-aortic node, and a final sample taken from a left fallopian tube lesion preserved in molecular fixative (total 8 areas of carcinoma and serum for germline comparison).

Case 6

Case 6 was a 65 year old of Dutch descent with a family history of pancreatic cancer in her mother, and no known breast or ovarian cancer in any family members. She had a personal history of carcinoid tumor of the terminal ileum diagnosed at age 63 and had undergone hemicolectomy but did not require additional therapy (e.g. no chemotherapy nor radiation administered). She presented with ascites and a pelvic mass 24 months later and it was assumed her carcinoid tumor had returned. Her CA125 level (900 kU/L) was elevated and the gynecologic oncology service

consulted for a planned joint procedure. At surgery she was found to have diffuse disease in both ovaries, multiple peritoneal deposits and ascites, omental caking, and a liver lesion. It was not deemed possible to optimally debulk this patient and priority was given to obtaining tissue to determine if her ovarian mass and omental disease were in fact carcinoid vs. a new primary. She underwent right salpingoophorectomy, omentectomy and core biopsy of the liver. Pathology revealed a poorly differentiated HGS carcinoma of the right ovary, fallopian tube, and omentum. Recurrent carcinoid disease was present in the liver. This patient was treated with chemotherapy for her ovarian cancer; IV CP for six cycles. She recurred 13 months after diagnosis (7 months after completion of chemotherapy) with pleural effusions (thoracentesis fluid confirmed ovarian adenocarcinoma) and was again treated with chemotherapy. She ultimately received a total of 21 cycles of chemotherapy, including CP and four different agents. She became symptomatic with bulky disease and decreased response to therapy two years later (42 months from primary surgery) and was therefore taken to surgery for extensive debulking, including removal of her remaining ovary (left ovary had been left in situ) and para-aortic lymph node debulking. HGS cancer was confirmed in the left ovary and para-aortic nodes. This patient died with recurrent ovarian carcinoma 74 months after diagnosis and surgery for her HGS cancer. No BRCA1/2 mutations were identified on germline testing. Samples for comparison included omental tumor at primary surgery (chemotherapy naive), and left ovary at recurrence.

3 Specimen preservation and histologic evaluation

In cases identified with high grade serous histology, multiple tissue samples were obtained from primary ovarian tumor and metastatic sites where adequate tumor volume permitted. When the ovary was pathologically enlarged, samplings were taken from up to five different areas with an effort made to equally space samples while staying within grossly apparent tumor tissue. Each sampling is cut into three pieces, yielding two end-pieces for cryovials and a middle portion placed in 10% buffered formalin. Fallopian tubes that appeared to be in good condition (identifiable, not obliterated by disease), were resected from the surgical specimen, measured (length and width), pinned onto a wax block, and placed in molecular fixative overnight. The isthmus, ampulla, infundibulum, and fimbriae were serially sectioned transversely (cross sections, perpendicular to the long-axis of the tube) at 1.5 mm intervals and placed into tissue cassettes. The sections were processed in Molecular Fixative overnight followed by processing in the Sakura Tissue-Tek Xpress Rapid Tissue Processor (Somagen Diagnostics) under controlled nuclease-free conditions (Turashvili et al., 2011). Molecular fixative (Somagen Diagnostics) is a mixture of methanol and polyethylene glycol (90% and 10%, respectively) (US patent number 7,138,226).

All paraffin-embedded blocks, including formalin-fixed tumor samples and molecular-fixed fallopian tubes, were sectioned and stained with hematoxylin and eosin prior to expert histopathological review to confirm the presence of high grade serous carcinoma. The co-existence of high-grade serous and mixed endometrioid-serous carcinoma in case 4 was confirmed by WT-1 immunohistochemistry of representative blocks from the left and right ovary. Aberrant TP53 protein expression was similarly assessed in these samples, in addition to the fallopian tube lesion used for sequencing, prior to coring for DNA extraction. Methods for WT-1 and TP53 immunohistochemistry are previously described by Kobel et al (Köbel et al., 2008).

The resultant blocks were sectioned and stained with hematoxylin and eosin, pathologically reviewed, and diagnostically confirmed for the presence of carcinoma.

4 Fallopian tube identification, coring and extraction

Multiple small areas of high grade serous carcinoma were identified in the left fallopian tubes of Case 4 and 5 by expert histopathologic review of H&E stained sections (Gilks). Although selected lesions for both cases appeared to be early, stromal invasion was observed, precluding a diagnosis of serous intraepithelial carcinoma. Corresponding areas were then identified in the corresponding paraffin-embedded blocks. Lesions were initially isolated from 5-10 x 7um sections using the Veritas Laser Capture Microdissection System followed by extraction of genomic DNA from captured cells using the PicoPure DNA Extraction Kit (both from Arcturus Biosciences, Inc). As this method resulted in insufficient DNA for our downstream experiments without the need for whole genome amplification, we elected to collect two x 0.6mm cores of >90% carcinoma cells from each case. DNA was extracted using Ambions Recoverall

Total Nucleic Acid Kit. All DNA quantification was performed using the Qubit 2.0 Fluorometer (Case 4j=25ng/uL; Case 5h=13.4ng/uL).

5 Fluorescent In Situ Hybridization

Locus specific FISH analysis was performed as previously described (Shah et al., 2009) using full 5 micron sections from representative FFPE blocks. Briefly, BACs were directly labeled with spectrum green or orange using a Nick Translation Kit (Abbott Molecular, Illinois, USA) and chromosomal locations were validated using normal metaphases from blood (results not shown). Specific BAC and control probe identifiers are listed in the corresponding figures. Nuclei were counterstained with 4,6- diamidino 2-phenylindole and signals and patterns were identified on a Zeiss Axioplan epifluorescent microscope and were scored manually in 80 nuclei using an oil immersion 100x objective. Images were captured using Metasystems software (MetaSystems Group Inc., Belmont, MA, USA) and an oil immersion 63x objective.

6 Sequencing and mutation identification

Exome sequence data generation

Genomic DNA was extracted from frozen samples using the Gentra Puregene kit (Qiagen) and quantified using a Qubit fluorometer (Invitrogen). Exome capture was achieved through solution hybrid selection with the Human All Exon kit SureSelect Target Enrichment System (Agilent) version 1 for Illumina Genome Analyzer paired-end sequencing (Gnirke et al., 2009) and libraries were prepared as described in (Morin et al., 2011). Total sequence generation per case, boxplots of coverage for exons for each sample can be found Table S1 and Figure S1.

Exome sequence alignment and mutation calling

Sequence reads were aligned to the human reference sequence (NCBI build 36, hg18) and a compacted reference composed of the targeted exons. Alignments were performed using Maq v0.7.1 (Li et al., 2008) using default parameters. Uniquely aligned reads were kept for downstream analysis. Using the resulting tumour and normal pair of BAM files, somatic SNVs were called by JointSNVMix algorithm (Roth et al., 2012), sorted by marginal posterior probability and further run through the mutationSeq algorithm (Ding et al., 2012) to find high confidence candidate SNVs. Non-synonymous and synonymous mutations were called using MutationAssessor (Reva et al., 2011) (http://mutationassessor.org). Indels were predicted using samtools v0.1.7 pileup command from BAM files. Indels present in at least 10% of reads covering the position of interest, predicted to affect a coding sequence, and for which there were 0 reads containing the same indel in the corresponding normal DNA library were carried forward for validation as candidate somatic indels.

Validation of mutations

Candidate SNVs and indels were subjected to deep sequencing using Illumina and Ion Torrent platforms (details bellow).

Deep amplicon sequencing using the Illumina GAII platform

Somatic mutation predictions were subjected to rigorous validation by targeted deep amplicon sequencing in the tumour and normal DNA templates. Genomic DNA was prepared as previously described (Shah et al., 2009) . Automated primer design was performed using Primer3 (Rozen et al., 2000) and custom scripting. Primer pairs were designed to place the variant position within 75bps of either end of the amplicon and to be between 50-300bp in length. Primer pairs were independently validated by in silco PCR followed by BLAT against the human genome to ensure that the correct target was generated and that the resulting amplicon was unique within the genome. DNA

primers were synthesized in 96-well plates at a 25nmol scale with standard desalting (IDT Coralville, IA USA). Polymerase cycling reactions were set up in 96-well plates and comprised of 0.5 μM forward primer, 0.5 μM reverse primer, 1-2 ng of gDNA template, 5X Phusion HF Buffer, 0.2 μM dNTPs, 3% DMSO, and 0.4 units of Phusion DNA polymerase (NEB, Ipswich, MA, USA). Reaction plates were cycled on a MJR Peltier Thermocycler (model PTC-225) with cycling conditions of a denaturation step at 98 °C for 30 sec, followed by 35 cycles of [98 °C for 10 sec, 69 °C for 15 sec, 72 °C for 15 sec] and a final extension step at 72 °C for 10 min. PCR reactions were visualized on 3% agarose (NuSieve) gels for 2hrs at 170V to assess PCR success. Successful reactions were manually pooled (4ul per well) by template and subjected to Illumina library construction using a modified paired-end protocol (Illumina, Hayward, USA). This involved A-tailing of the amplicons and ligation to Illumina PE adapters. Adapter-ligated products were purified on Qiaquick spin columns (Qiagen, Valencia, CA, USA) and PCR-amplified using Phusion DNA polymerase (NEB, Ipswich, MA, USA) in 10 cycles using PE primer 1.0 (Illumina) and a custom multiplexing PCR Primer [5-CAAGCAGAAGACGGCATACGAGAT NNNNNNCGGTCTCGGCATTCCTGC TGAACCGCTCTTCCGATCT-3] where *NNNNNN* was replaced with unique fault tolerant hexamer barcodes for each template. PCR products of the desired size range were purified away from adapter ligation artifacts using 8% PAGE gels, pooled and DNA quality was assessed and quantified using an Agilent DNA 1000 series II assay (Agilent, Santa Clara CA, USA) and Nanodrop 7500 spectrophotometer (Nanodrop, Wilmington, DE, USA) and subsequently diluted to 10nM. The final concentration was confirmed using a Quant-iT dsDNA HS assay kit and Qubit fluorometer (Invitrogen, Carlsbad, CA, USA). For sequencing, clusters were generated on the Illumina cluster station using v4 cluster reagents and paired-end 75bp reads generated using v4 sequencing reagents on the Illumina GAII platform following the manufacturer's instructions. Between the paired 75bp reads a third 7 base pair read was performed using the following custom sequencing primer [5- GATCGGAAGAGCGGTTCAGCAGGAATGCCGAGACCG] to sequence the hexamer barcode. Image analysis, base-calling and error calibration was performed using v1.60 of Illumina's Genome analysis pipeline.

Deep amplicon sequencing using the Illumina MiSeq platform for cell-free DNA in the plasma

Circulating tumour DNA was extracted from 1-2 ml of plasma as per the Qiagen Circulating Nucleic Acid Kit directions. Plasma was obtained from centrifuged blood collected in EDTA collection tubes and frozen at -80C.

The primers used and the PCR cycling conditions were the same as in the previous section. Circulating DNA was used as PCR templates at 0.1ng per reaction. Illumina libraries were constructed on the SPRI-TE Nucleic Acid Extractor (Beckman Coulter) using the SPRIworks Fragment Library System I with no on-board size selection. PE adapter-ligated products were cleaned using 1.8 volumes of Agencourt AMPure XP beads (Beckman Coulter) and PCR-amplified using Phusion DNA polymerase (NEB, Ipswich, MA, USA) in 10 cycles using PE primer 1.0 (Illumina, Hayward, USA) and a custom multiplexing PCR primer [5-CAAGCAGAAGACGGCATACGAGATNNNNNNCGGTCTCGGCATTCCTGCTGAACCGCTCTTCCGATCT-3] where NNNNNN was replaced with unique fault tolerant hexamer barcodes for each template. PCR products of the desired size range were purified away from adapter ligation artifacts using 8% PAGE gels. DNA quality was assessed using an Agilent DNA 1000 series II assay (Agilent, Santa Clara CA, USA) and quantified using a Qubit spectrophotometer (Invitrogen), and libraries were pooled and diluted to 8nM. 600ul of 11pM pooled library was sequenced on an upgraded Illumina MiSeq with paired-end 250bp reads using v2 reagents as per the manufacturer's instructions. A third 7bp read was performed using the custom index read primer [5- GATCGGAAGAGCGGTTCAGCAGGAATGCCGAGACCG] to sequence the hexamer barcode. An in-house generated PhiX control library was spiked in to the sample at 1% molar ratio as a sequencing control.

Deep amplicon sequencing using the Ion Torrent PGM platform

Sequence validation using custom primer panels

Validations of candidate single nucleotide variants (SNVs) identified from the initial sequencing of the primary ovarian cancer samples were achieved using a deep amplicon sequencing approach. Candidate SNVs were fed into the LIFE Technology AmpliSeq custom designer pipeline, after conversion to hg19 coordinates, and three multiplex primers pools were generated. The primers were designed to generate 100bp amplicons to accommodate the sequencing of DNA from formalin-fixed paraffin-embedded (FFPE) tissues. Pooled and unpooled primers, based on these designs, were purchased from IDT Technology (Coralville, Iowa, USA). Genomic DNA from the primary tumours, secondary

tumour nodules, metastatic lymph nodes and blood were harvested from snap- frozen tissues using the Qiagen Gentra Puregene kit (Valencia, CA, USA). Genomic DNA from the precursor lesions of samples DG1008 and DG1009 were extracted using the Ambion RecoverAll kit (Life Technologies, Burlington, ON, Canada) from macrodissected tissue isolated from FFPE block after spatial confirmation on matching hematoxylin/eosin- stained slides. All DNA specimens were quantitated using the QUBIT high sensitivity DNA assay kit (Life Technologies). Three separate multiplex amplicon PCR reactions were used for each tissue sample to cover all three amplicon pools using the Ion AmpliSeq Kit 2.0 Beta (Life Technologies). Each amplicon library (125-275 base pair) was quantitated using the High sensitivity DNA chip on the Agilent BioAnalyzer (Santa Clara, CA, USA). The three amplicon libraries of individual tissue samples, except for the two precursor lesions, were pooled for sequencing. Each individual amplicon library generated from the precursor lesions was sequenced separately in order to achieve deeper coverage.

Emulsion PCR (emPCR) libraries were generated on the Ion Torrent OneTouch platform using the Ion OneTouch 200bp Template kit (Life Technologies). The libraries were enriched using the Ion Torrent ES platform and all sequencing were done using the Ion PGM 200 Sequencing kit on 100 Megabase chip (Ion 316) on the Ion Torrent PGM platform.

AmpliSeq Cancer panel sequencing of selected tumour samples

AmpliSeq Cancer panel sequencing of selected tumour samples Samples from multiple tumour sites and blood of Case 4 and Case 5 were additionally sequenced using the AmpliSeq Cancer panel (Life Technologies) which covers 739 potential mutations in 46 genes. Amplicon libraries were indexed using the Ion Xpress Barcode Adapter kit and quantitated using the High sensitivity DNA chip on the Agilent BioAnalyzer (Santa Clara, CA, USA). Appropriate dilutions were performed based on amplicon concentration at the 130-210 basepair range. Indexed amplicon libraries were pooled for emulsion PCR and sequencing on the Ion Torrent PGM platform using the 316 and 318 chip.

Validation data analysis

Using Maq (v0.7.1) aligner (Li et al., 2008), 75bp paired end illumina reads were aligned to a reference database containing only the targeted loci for each case. Single end Ion Torrent PGM reads were aligned to a reference database containing only the targeted loci for each case using the BWA-SW algorithm (Li and Durbin, 2010) implemented in BWA (v0.5.9).

For each targeted sequence, we inferred the presence/absence of the targeted variants using a Binomial exact test as previously described (Shah et al., 2009). In addition, we imposed the following criteria in order to classify a 'validated' somatic mutation:

- both tumour and normal data had a minimum of 50 reads covering the targeted position
- the Binomial exact test result (Benjamini Hochberg adjusted p-value) for the tumour < 0.01
- the Binomial exact test result (Benjamini Hochberg adjusted p-value) for the normal $>= 0.01$
- the proportion of reads indicating the variant in the tumour $\geq 5\%$

Indels were called by the Samtools pileup command. To get better call results, the Illumina data were realigned by a gapped aligner GSNAP (Wu and Nacu, 2010), and then Samtools was used to call indels. For Ion Torrent PGM data, the GSNAP aligner didn't improve the indels calls, so we used the BWA-SW alignment results to call indels. For indels, we required:

- a minimum of 10% of all reads aligned to the position containing the indel
- at least 3 reads containing the indel
- a minimum local realignment score of 300

7 Affymetrix SNP6 analyses

Copy number analysis of SNP6 genotyping array samples were performed as described previously in Shah et al. (2012).

Normalization of intensities

We used two normalization techniques: PennCNV-Affy (Wang et al., 2007) and CRMAv2 (Bengtsson et al., 2009).

PennCNV-Affy produced normalized input log ratios and B-allele frequencies from the 27 tumour and 6 matched normal Affymetrix SNP6 samples. The normalization steps were performed as described in `http://www.openbioinformatics.org/penncnv/penncnv_tutorial_affy_gw6.html` whereby the tumour samples and the normal samples were batched separately. These results were used as input to OncoSNP for downstream analyses.

CRMAv2 produced independent, single-array normalized intensity data for 27 tumour and 6 normal samples. We applied the default settings using the following tags: ACC,ra,-XY,BPN,-XY,AVG,A+B,FLN,-XY. For each sample, allelic-crosstalk calibration, probe sequence effects normalization, probe-level summarization, and PCR fragment length normalization were performed. The following annotation files were used (Feb 14, 2008): Chip definition file (GenomeWideSNP_6.Full.cdf), Unit fragment-length (GenomeWideSNP_6,Full,na24,HB20080214.ufl), Unit genome position (GenomeWideSNP_6,Full,na24,HB20080214.ugp). Log ratios were computed for both tumour and normal arrays by normalizing each array independently against a common reference. We generated a "masked" reference from the HapMap270 (International HapMap Consortium et al., 2007) dataset that was free of inherent copy number polymorphisms, while accounting for systematic biases; this is described in Shah et al. (2012). The results of this normalization were used as input to HMM-Dosage for downstream analyses.

Copy number and loss of heterozygosity analysis of Affymetrix SNP6.0

We determined regions of copy number changes and loss of heterozygosity (LOH) in 27 tumour samples (across 6 patients) by analyzing the Affymetrix SNP6 arrays using the OncoSNP software v1.1 (May 31, 2011 release) (Yau et al., 2010). The paired tumour-normal OncoSNP analysis was used because matched normals for all 6 patients were available. This produced copy number results for the tumours but not for the matched normals themselves (we used another method for this, see below). We used the provided configurations and hyperparameters, specific to the Affymetrix platform, for this analysis. Other OncoSNP settings included using the following flags: "–subsample 30 –emiters 15 –stromal –intratumor". Two to 6 copy 'Somatic' LOH predictions made by OncoSNP were consolidated as simply LOH; 'Mono-allelic amplification' states were interpreted as 'Allele-specific copy number amplifications' (ASCNA). We also simplified discrete copy number predictions by occasionally referring to 3 copies as gain and 4 to 8 copies as amplification.

OncoSNP segments were post-processed for each tumour sample by converting CNA segments that had at least 10% reciprocal genomic overlap (base pairs) with germline CNVs in the corresponding matched normal sample.

Analysis of extreme copy number events in Affymetrix SNP6 HGS samples

For extreme copy number events (e.g. homozygous deletions and high-level amplifications), we performed copy number analysis on 34 SNP6 arrays, consisting of 27 total tumours samples and one matched-normal sample for each of the 6 patients. We employed HMM-Dosage (Curtis et al., 2012) (`http://compbio.bccrc.ca/software/hmm-dosage/`), which is a modified version of CNA-HMMer (Shah et al., 2006) designed to detect and distinguish the somatic and germline copy number events in cancer genomes interrogated by SNP array data. We chose this tool because accounting for germline events may help improve model accuracy in detecting somatic events. This model uses a state-space of 11 biologically interpretable classes: 5 somatic CNA states (K_{CNA}), 5 germine CNV states

(K_{CNV}), and the copy neutral state,

$$\begin{aligned} K_{CNV} &= \{CNVHOMD, CNVHETD, CNVGAIN, CNVAMP, CNVHLAMP\} \\ K_{CNA} &= \{HOMD, HETD, NEUT, GAIN, AMP, HLAMP\} \end{aligned} \quad (1)$$

HMM-Dosage requires prior CNV information which was computed as probe-level CNV frequencies found in the following combined datasets: 482 normal samples from METABRIC (Curtis et al., 2012) and an external dataset of 450 HapMap samples whose CNVs were predicted by Conrad et al. (2010). See Curtis et al. (2012) and Shah et al. (2012) for more information. Initial parameters of the Student's-t emission means used in the HMM-Dosage analysis were $log2\big([1.0987, 1.4063, 1.9790, 2.7608, 3.7022, 4.9647]/2\big)$ for K_{CNA} states and $log2\big([0.5, 1, 3, 5, 7]/2\big)$ for K_{CNV} states.

For patient-specific germline CNVs that are not found in the prior information, post-processing of the segments were also performed similar to OncoSNP segments. Results from this was used exclusively for extreme copy number analysis while all other copy number analyses used results from OncoSNP described above.

Gene alterations

Identification of genes affected by copy number and LOH was performed similarly to previous studies (Curtis et al., 2012; Shah et al., 2012). The gene annotations and coordinates were extracted from Ensembl 54 (hg18) and protein-coding genes, totalling to 20878 unique genes, were selected for analysis. Genes overlapping homozygous deletions and high-level amplifications were also compared to the Cancer Gene Census (version date 2011-11-15) (Futreal et al., 2004).

8 Clonal frequency analysis

We used a hierachical Bayesian model which accounts for uncertainty about genotype to estimate the frequency of cells with a given mutation. We imagine each mutation divides the population of cells into two classes; those without the mutation we call the reference population and those with we refer to as the variant population. We treat the reference population as though it has fixed genotype of *AA*. We model the genotype of the variant population at a given locus using one of 27 states allowing for copy numbers from 1-6 and differing zygosity status. We use information about copy number and LOH status derived from OncoSNP (Yau et al., 2010) analysis to inform the prior beliefs on the genotype of the variant population. To allow multiple mutations to have the same frequency, we use a Dirichlet process prior on the class frequencies. Sites which are clustered by this model have cellular frequencies which are sufficiently similar that they are better explained as originating from a single class of cells. Inference for this model is performed using Markov chain Monte Carlo sampler.

The full hierarchical Bayesian model presented as a probabilistic graphical model is shown in Figure S13. Input to the model is a set of mutations and their allele abundance (reference, variant). In addition, the copy number and LOH status at the position of each mutation is given. The output of the model is a mutation co-occurrence matrix containing the proportion of MCMC samples in which each pair of mutations were grouped together, and the clonal frequency estimation for each of the MCMC samples, thus allowing inference of a posterior distribution of clonal frequency estimates.

Software implementing this approach called *PyClone* (Roth et al., manuscript in preparation) is available upon request from the authors. All samples except for the fallopian tube lesions for which copy number data were not available were run through the clonal analysis pipeline. Clonal frequency estimates for each case were first adjusted by normalizing by the maximum clonal frequency value to remove the effect of normal cell contamination. Clonal frequency distributions for each mutation were then plotted for all the samples of a case. Mutations with high clonal frequencies are thought to be early events in cancer development.

9 Affymetrix HT-HG-U133A 2.0

9.1 Sample preparation

RNA for all samples (except for the fallopian tube) was extracted using the miRNeasy Kit (Qiagen) and run on GeneChip Human Genome U133A 2.0 Array (Affymetrix) as per the manufacturer's instructions. RNA quantity and quality were analyzed using the Agilent 2100 Bioanalyser Nanochip (Agilent Technologies).

9.2 Data processing

Expression data for all 29 samples were preprocessed using RMA and quantile normalization at the probe level (affy Bioconductor package `http://www.bioconductor.org/packages/2.11/bioc/html/affy.html`). Since these samples were run in five different batches, we used COMBAT to correct for batch effects on the probe-level (sva Bioconductor package (Leek et al., 2012)), mapped the probes to genes and summarized the probeset expression on the genes. Affymetrix HG U133 plus 2 CEL files of 594 TCGA HGSOC patients were downloaded from the TCGA portal (`https://tcga-data.nci.nih.gov/tcga/`) and were preprocessed in an identical fashion that our samples were analyzed (explained above, COMBAT batch correction with 13 batches). Another level of batch correction was performed when data were combined with the expression data of the 594 TCGA samples. To assure that the batch correction scheme has worked, we clustered TCGA expression samples before and after batch correction (using k-Medoids clustering approach with k=5 (Kaufman and Rousseeuw, 1987)) and did not see any significant changes in the cluster memberships before vs. after batch correction (adjusted rand index=0.986 (Rand, 1971)) Mean row subtraction of log transformed data was applied, and the 1,500 most variable genes were selected, using a median absolute deviation (MAD) filter, for subsequent hierarchical clustering of the expression profiles.

10 Phylogenetic analysis

Phylogenetic trees were generated based on three different genomic events: mutations data, copy number and compound copy number events. For mutation data, a consolidated matrix containing the mutations of all samples (rows) with '1' and '0' representing the presence and absence of a mutation in a gene (column), respectively, is generated. The rows of this matrix represent the samples and columns represent the genes. For copy number, the matrix consisted of segment log ratio data for each patient-gene pair. Pearson correlation coefficients ρ_{xy} were computed between pairs of patients x and y. The results were used for phylogenetic analysis such that the pairwise distance of x and y was computed as $1 - \rho_{xy}$. The Neighbor-Joining method of Saitou and Nei (Saitou and Nei, 1987) and the Unweighted Pair Group Method with Arithmetic Mean (UPGMA) method of clustering were used to construct the phylogenetic tree. We used 'ape' R package (Paradis et al., 2004) for constructing and plotting the phylogenetic trees.

For compound copy number events, a similar procedure was performed with the exception of the matrix construction and computation of the distance. The matrix consisted of the weight of observing compound events: 2 for amplified LOH (ALOH), 2 for copy neutral LOH (NLOH), 2 for homozygous deletion (HOMD), 1 for hemizygous deletion (HETD), and zero for diploid heterozygous (HET) and allele-specific amplification (ASCNA). Euclidean distance was computed between pairs of tumour samples. The tree construction was performed the same as before.

References

Bengtsson, H., Wirapati, P., and Speed, T. P., 2009. A single-array preprocessing method for estimating full-resolution raw copy numbers from all affymetrix genotyping arrays including genomewidesnp 5 & 6. *Bioinformatics*, **25**(17):2149–56.

Conrad, D. F., Pinto, D., Redon, R., Feuk, L., Gokcumen, O., Zhang, Y., Aerts, J., Andrews, T. D., Barnes, C., Campbell, P., *et al.*, 2010. Origins and functional impact of copy number variation in the human genome. *Nature*, **464**(7289):704–12.

Curtis, C., Shah, S. P., Chin, S. F., Turashvili, G., Rueda, O. M., Dunning, M. J., Speed, D., Lynch, A. G., Samarajiwa, S., Yuan, Y., *et al.*, 2012. The genomic and transcriptomic architecture of 2,000 breast tumours reveals novel subgroups. *Nature*, **486**(7403):346–352.

Ding, J., Bashashati, A., Roth, A., Oloumi, A., Tse, K., Zeng, T., Haffari, G., Hirst, M., Marra, M., Condon, A., *et al.*, 2012. Feature-based classifiers for somatic mutation detection in tumour–normal paired sequencing data. *Bioinformatics*, **28**(2):167–175.

Futreal, P. A., Coin, L., Marshall, M., Down, T., Hubbard, T., Wooster, R., Rahman, N., and Stratton, M. R., 2004. A census of human cancer genes. *Nat Rev Cancer*, **4**(3):177–183.

Gnirke, A., Melnikov, A., Maguire, J., Rogov, P., LeProust, E. M., Brockman, W., Fennell, T., Giannoukos, G., Fisher, S., Russ, C., *et al.*, 2009. Solution hybrid selection with ultra-long oligonucleotides for massively parallel targeted sequencing. *Nat Biotechnol*, **27**(2):182–189.

International HapMap Consortium, Frazer, K. A., Ballinger, D. G., Cox, D. R., Hinds, D. A., Stuve, L. L., Gibbs, R. A., Belmont, J. W., Boudreau, A., Hardenbol, P., *et al.*, 2007. A second generation human haplotype map of over 3.1 million snps. *Nature*, **449**(7164):851–61.

Kaufman, L. and Rousseeuw, P., 1987. Clustering by means of medoids. .

Köbel, M., Kalloger, S. E., Boyd, N., McKinney, S., Mehl, E., Palmer, C., Leung, S., Bowen, N. J., Ionescu, D. N., Rajput, A., *et al.*, 2008. Ovarian carcinoma subtypes are different diseases: implications for biomarker studies. *PLoS Med*, **5**(12).

Leek, J. T., Johnson, W. E., Parker, H. S., Jaffe, A. E., and Storey, J. D., 2012. The sva package for removing batch effects and other unwanted variation in high-throughput experiments. *Bioinformatics*, **28**(6):882–883.

Li, H. and Durbin, R., 2010. Fast and accurate long-read alignment with burrows–wheeler transform. *Bioinformatics*, **26**(5):589–595.

Li, H., Ruan, J., and Durbin, R., 2008. Mapping short dna sequencing reads and calling variants using mapping quality scores. *Genome research*, **18**(11):1851–1858.

McAlpine, J. N., Porter, H., Köbel, M., Nelson, B. H., Prentice, L. M., Kalloger, S. E., Senz, J., Milne, K., Ding, J., Shah, S. P., *et al.*, 2012. Brca1 and brca2 mutations correlate with tp53 abnormalities and presence of immune cell infiltrates in ovarian high-grade serous carcinoma. *Mod Pathol*, **25**(5):740–750.

Morin, R., Mendez-Lago, M., Mungall, A., Goya, R., Mungall, K., Corbett, R., Johnson, N., Severson, T., Chiu, R., Field, M., *et al.*, 2011. Frequent mutation of histone-modifying genes in non-hodgkin lymphoma. *Nature*, **476**(7360):298–303.

Paradis, E., Claude, J., and Strimmer, K., 2004. Ape: analyses of phylogenetics and evolution in r language. *Bioinformatics*, **20**(2):289–290.

Rand, W. M., 1971. Objective criteria for the evaluation of clustering methods. *Journal of the American Statistical association*, **66**(336):846–850.

Reva, B., Antipin, Y., and Sander, C., 2011. Predicting the functional impact of protein mutations: application to cancer genomics. *Nucleic Acids Research*, **39**(17):e118–e118.

Roth, A., Morin, R., Ding, J., Crisan, A., Ha, G., Giuliany, R., Bashashati, A., Hirst, M., Turashvili, G., Oloumi, A., *et al.*, 2012. Jointsnvmix: A probabilistic model for accurate detection of somatic mutations in normal/tumour paired next generation sequencing data. *Bioinformatics*, .

Rozen, S., Skaletsky, H., et al., 2000. Primer3 on the www for general users and for biologist programmers. *Methods Mol Biol*, **132**(3):365–386.

Saitou, N. and Nei, M., 1987. The neighbor-joining method: a new method for reconstructing phylogenetic trees. *Molecular biology and evolution*, **4**(4):406–425.

Schrader, K., Hurlburt, J., S.E., K., Hansford, S., Young, S., Huntsman, D., Gilks, C., and McAlpine, J., 2012. Germline brca1 and brca2 mutations in ovarian cancer: Utility of a histology-based referral strategy. *Obstetrics and gynecology (Epub ahead of print)*, .

Shah, S., Morin, R., Khattra, J., Prentice, L., Pugh, T., Burleigh, A., Delaney, A., Gelmon, K., Guliany, R., Senz, J., *et al.*, 2009. Mutational evolution in a lobular breast tumour profiled at single nucleotide resolution. *Nature*, **461**(7265):809–813.

Shah, S. P., Roth, A., Goya, R., Oloumi, A., Ha, G., Zhao, Y., Turashvili, G., Ding, J., Tse, K., Haffari, G., *et al.*, 2012. The clonal and mutational evolution spectrum of primary triple-negative breast cancers. *Nature*, **486**(7403):395–399.

Shah, S. P., Xuan, X., DeLeeuw, R. J., Khojasteh, M., Lam, W. L., Ng, R., and Murphy, K. P., 2006. Integrating copy number polymorphisms into array cgh analysis using a robust hmm. *Bioinformatics*, **22**(14):e431–9.

Turashvili, G., Yang, W., McKinney, S., Kalloger, S., Gale, N., Ng, Y., Chow, K., Bell, L., Lorette, J., Carrier, M., *et al.*, 2011. Nucleic acid quantity and quality from paraffin blocks: Defining optimal fixation, processing and dna/rna extraction techniques. *Experimental and Molecular Pathology*, .

Wang, K., Li, M., Hadley, D., Liu, R., Glessner, J., Grant, S. F., Hakonarson, H., and Bucan, M., 2007. Penncnv: an integrated hidden markov model designed for high-resolution copy number variation detection in whole-genome snp genotyping data. *Genome Res*, **17**(11):1665–1674.

Wu, T. and Nacu, S., 2010. Fast and snp-tolerant detection of complex variants and splicing in short reads. *Bioinformatics*, **26**(7):873–881.

Yau, C., Mouradov, D., Jorissen, R. N., Colella, S., Mirza, G., Steers, G., Harris, A., Ragoussis, J., Sieber, O., and Holmes, C. C., *et al.*, 2010. A statistical approach for detecting genomic aberrations in heterogeneous tumor samples from single nucleotide polymorphism genotyping data. *Genome Biol*, **11**(9).
