# Supplementary figures and images for: Distinct evolutionary trajectories of primary high-grade serous ovarian cancers revealed through spatial mutational profiling
Source: J Pathol. 2013 Aug 6;231(1):21–34. doi: 10.1002/path.4230 (PMC3864404; doi:10.1002/path.4230)

Fig. S1

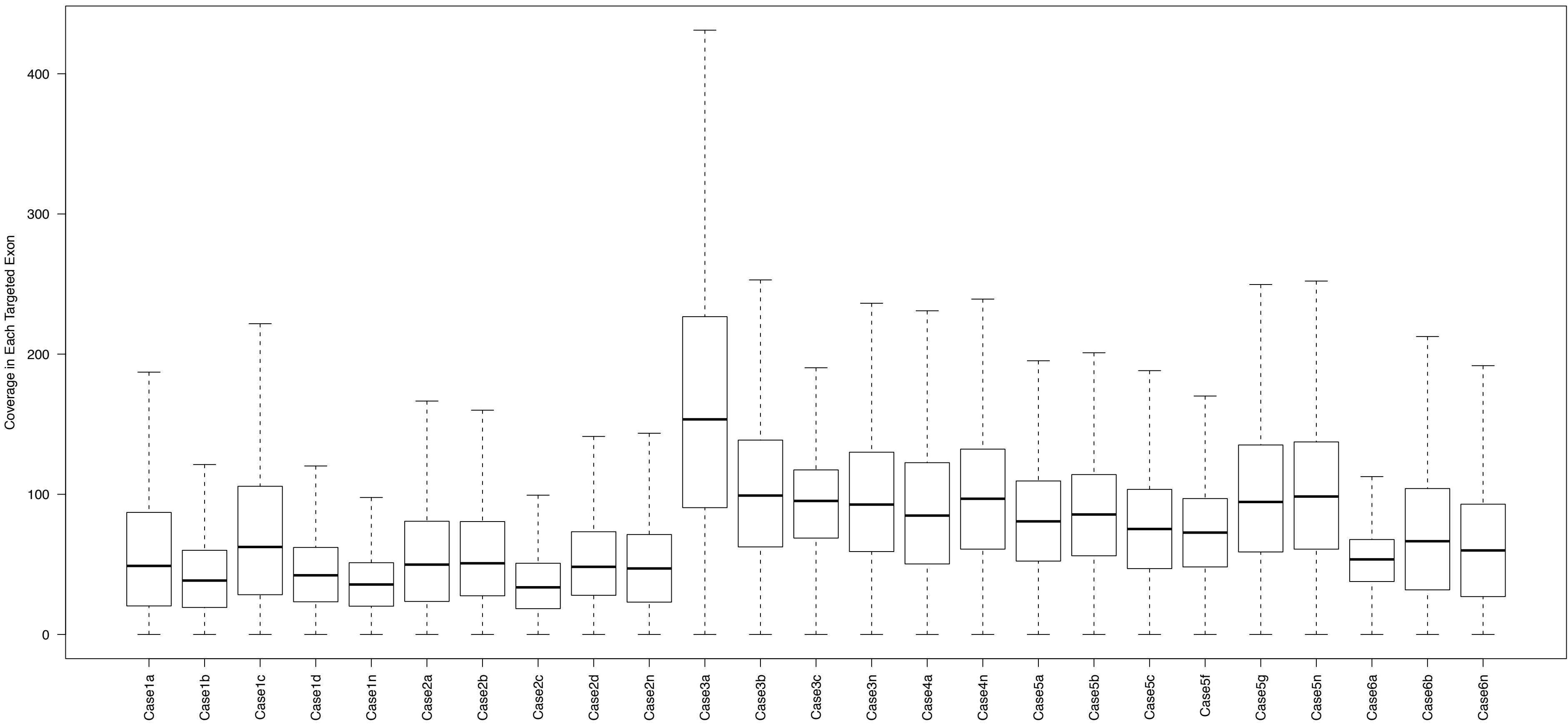

Supplement: Supplementary file 2 — Figure S2. Intratumoural mutational profile for case 1. [file path0231-0021-sd2.pdf]

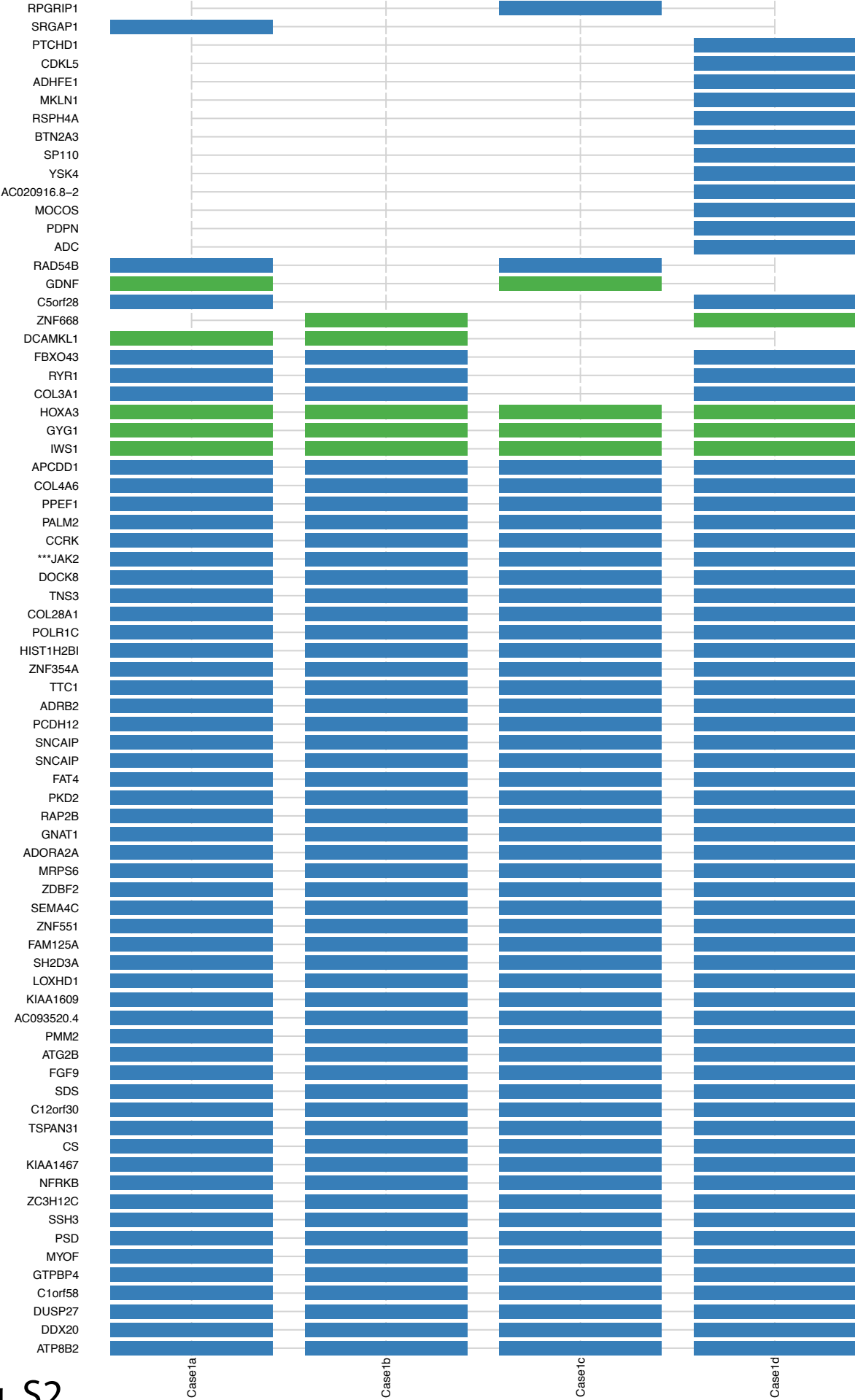

Fig. S2

Supplement: Supplementary file 3 — Figure S3. Intratumoural mutational profile for case 2. [file path0231-0021-sd3.pdf]

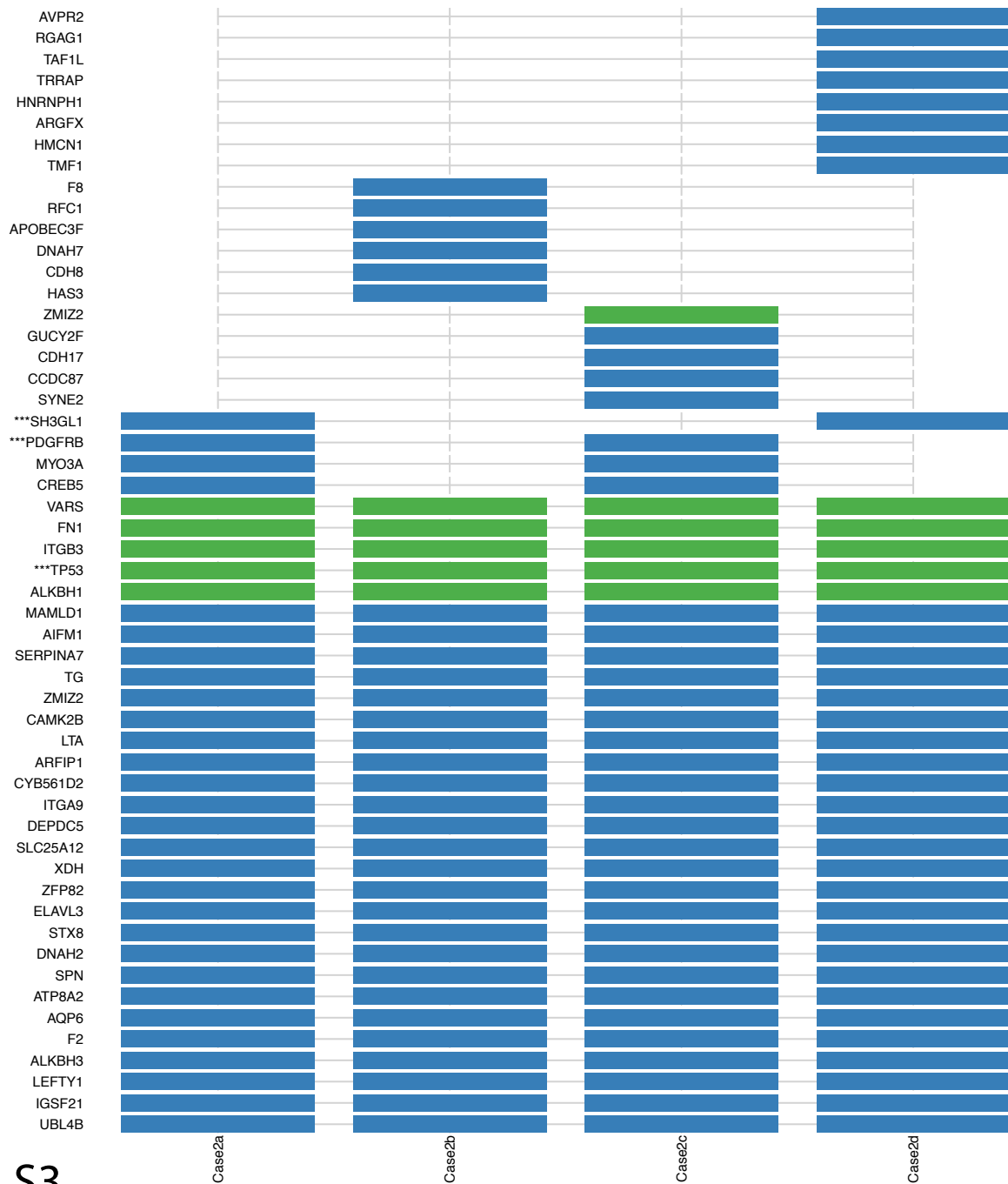

Fig. S3

Supplement: Supplementary file 4 — Figure S4. Intratumoural mutational profile for case 3. [file path0231-0021-sd4.pdf]

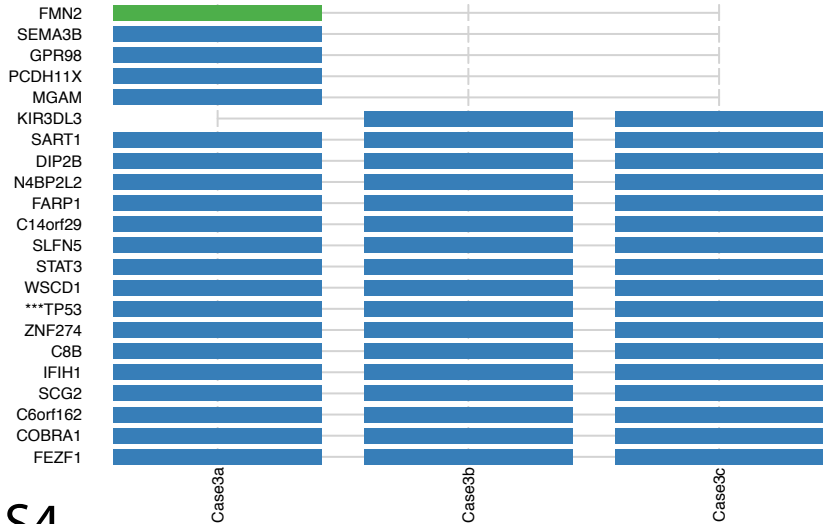

Fig. S4

Supplement: Supplementary file 5 — Figure S5. Intratumural mutational profile for case 4. [file path0231-0021-sd5.pdf]

Fig. S5

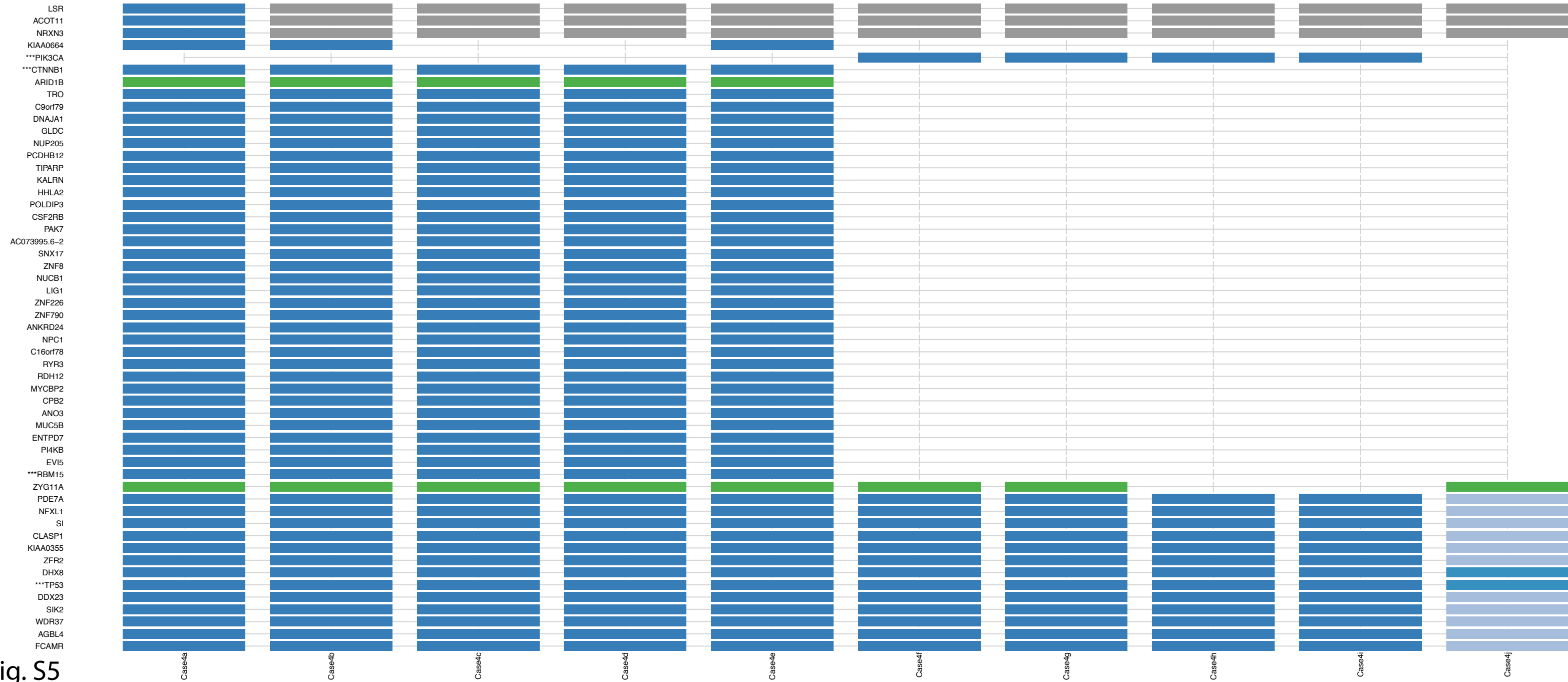

Supplement: Supplementary file 6 — Figure S6. Intratumoural mutational profile for case 5. [file path0231-0021-sd6.pdf]

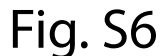

Supplement: Supplementary file 7 — Figure S7. Intratumoural mutational profile for case 6. [file path0231-0021-sd7.pdf]

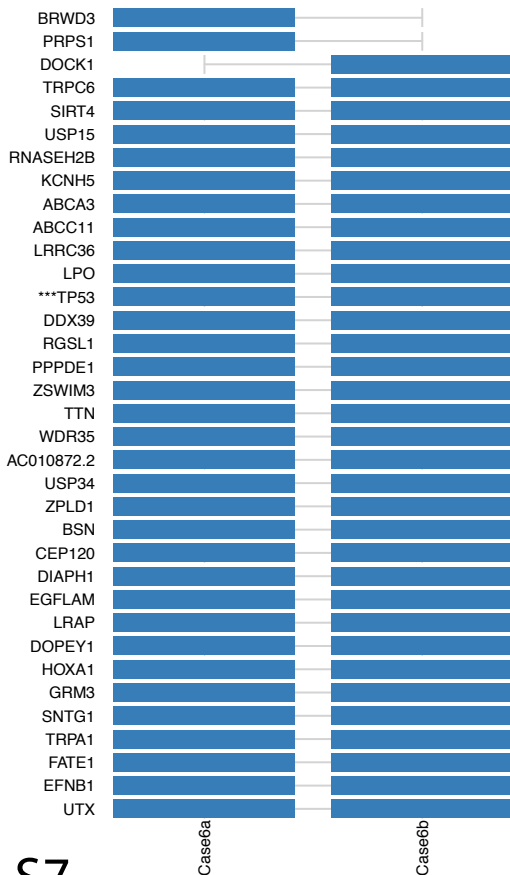

Fig. S7

Supplement: Supplementary file 8 — Figure S8. Immunohistochemistry (IHC) profiles for case 4 indicate mixed histology. [file path0231-0021-sd8.pdf]

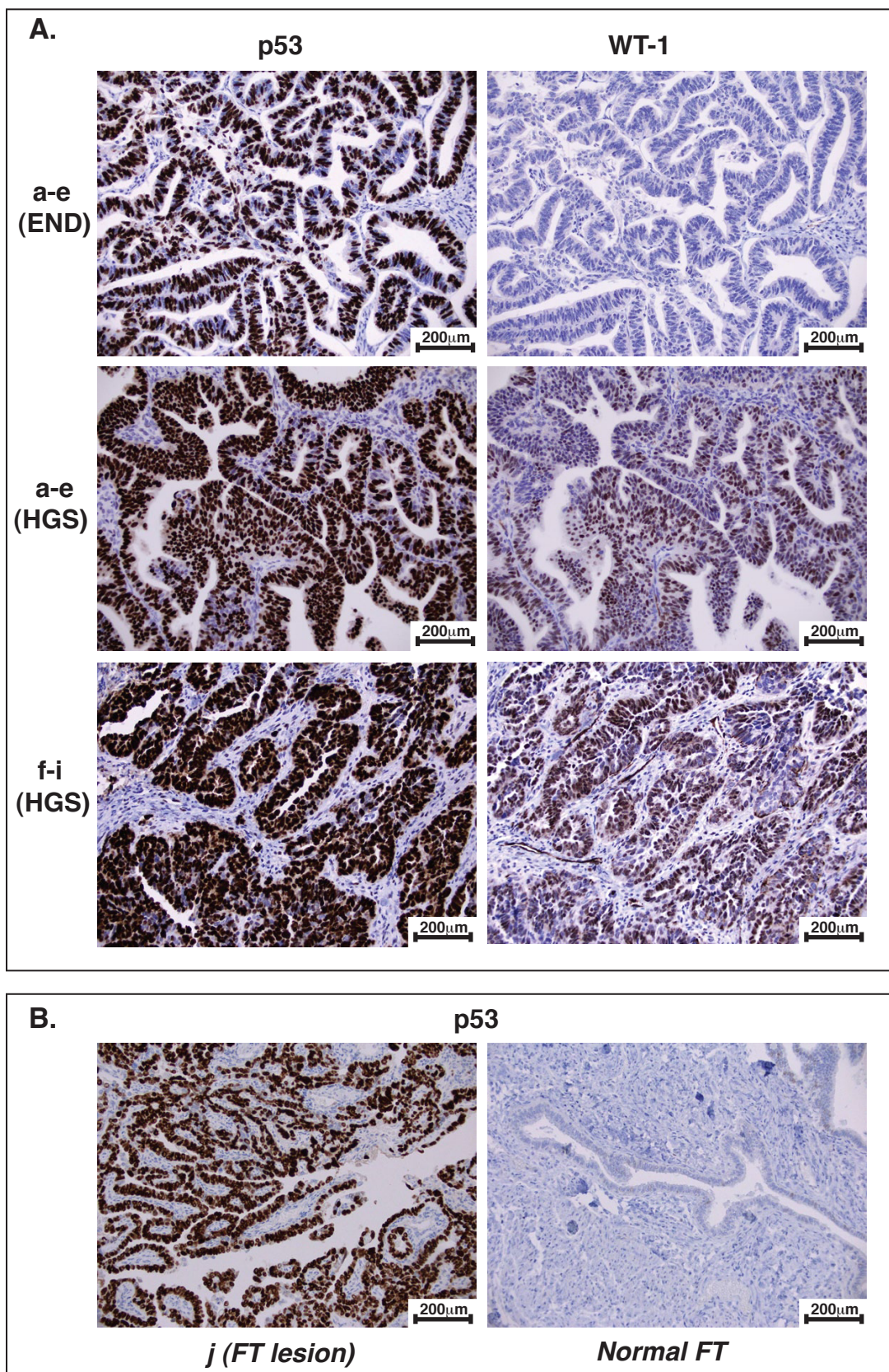

Fig. S8

Supplement: Supplementary file 9 — Figure S9. Genomic copy number architecture of intra-patient samples using Circos. [file path0231-0021-sd9.pdf]

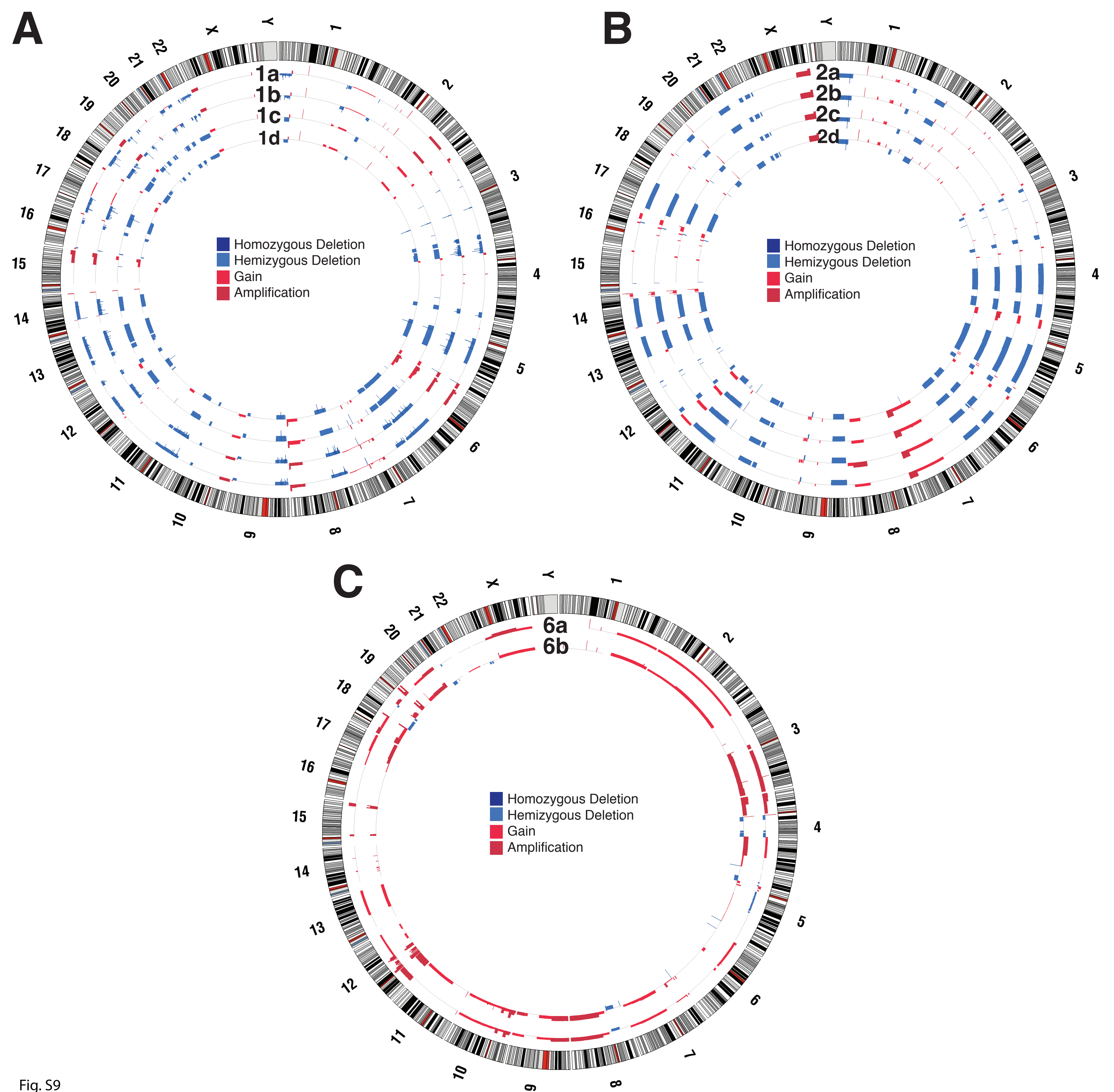

Fig. S9

Supplement: Supplementary file 10 — Figure S10. Copy number alteration and fluorescence in situ hybridization comparisons between right and left ovaries and fallopian tube of case 4 at chromosomes 6 and 12. [file path0231-0021-sd10.pdf]

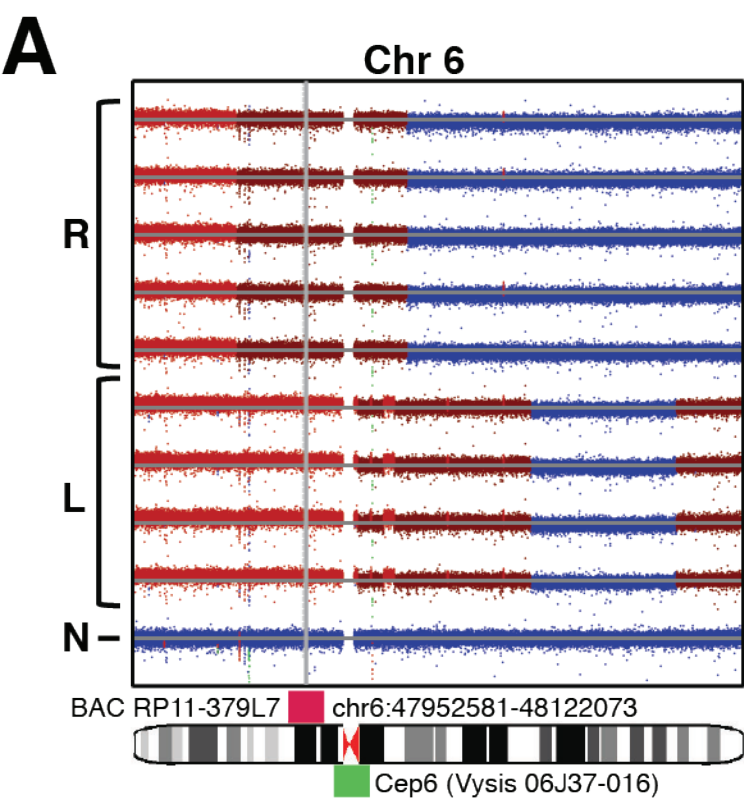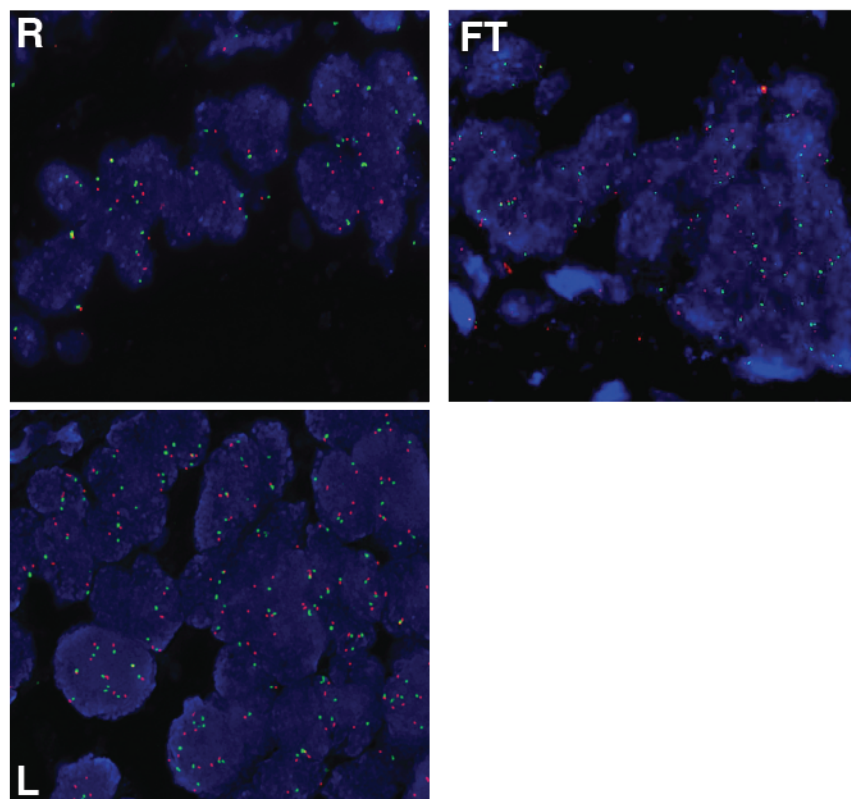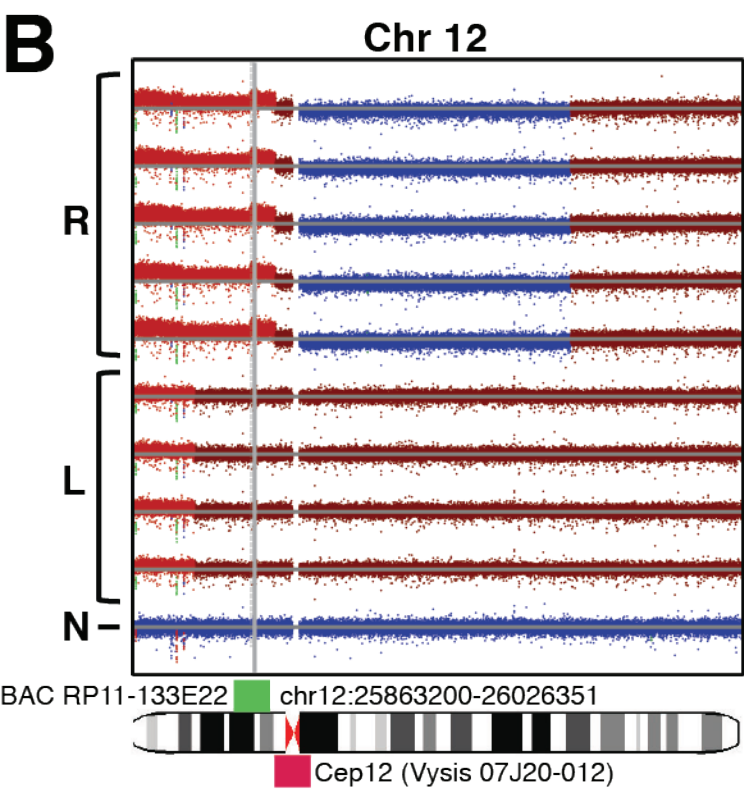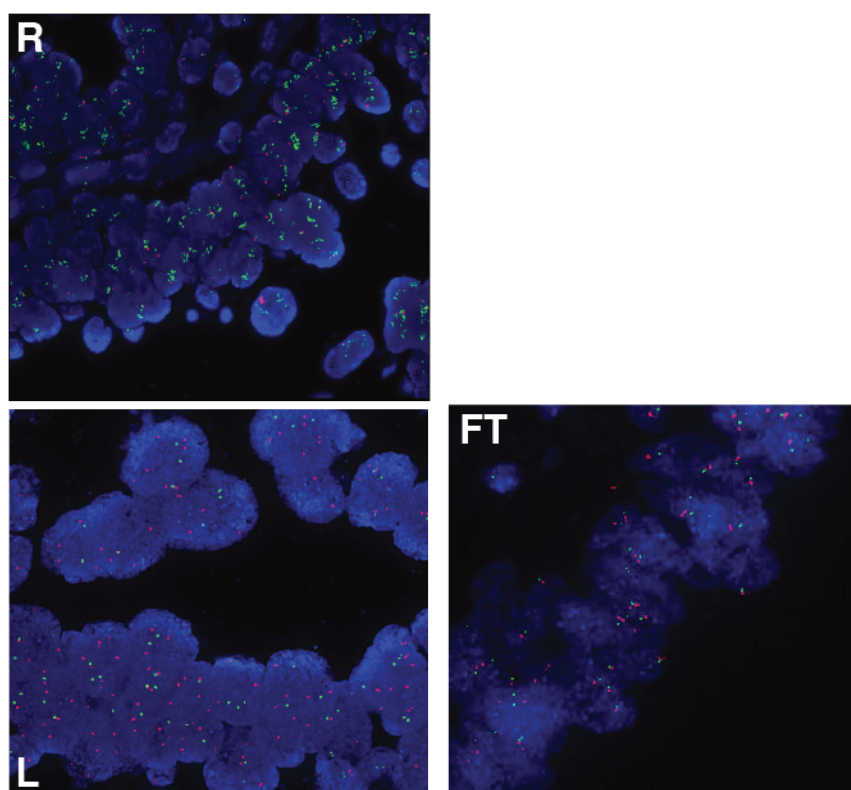

Fig. S10

Supplement: Supplementary file 11 — Figure S11. Heterogeneous NF1 homozygous deletions in cases 1 and 3. [file path0231-0021-sd11.pdf]
