# Supplementary material for: Distinct evolutionary trajectories of primary high-grade serous ovarian cancers revealed through spatial mutational profiling
Source: J Pathol. 2013 Aug 6;231(1):21–34. doi: 10.1002/path.4230 (PMC3864404; doi:10.1002/path.4230)
Supplement: Supplementary file 12 — Figure S12. Comparison of cases 3a and 3b reveals genome doubling. [file path0231-0021-sd12.pdf]

Fig. S11

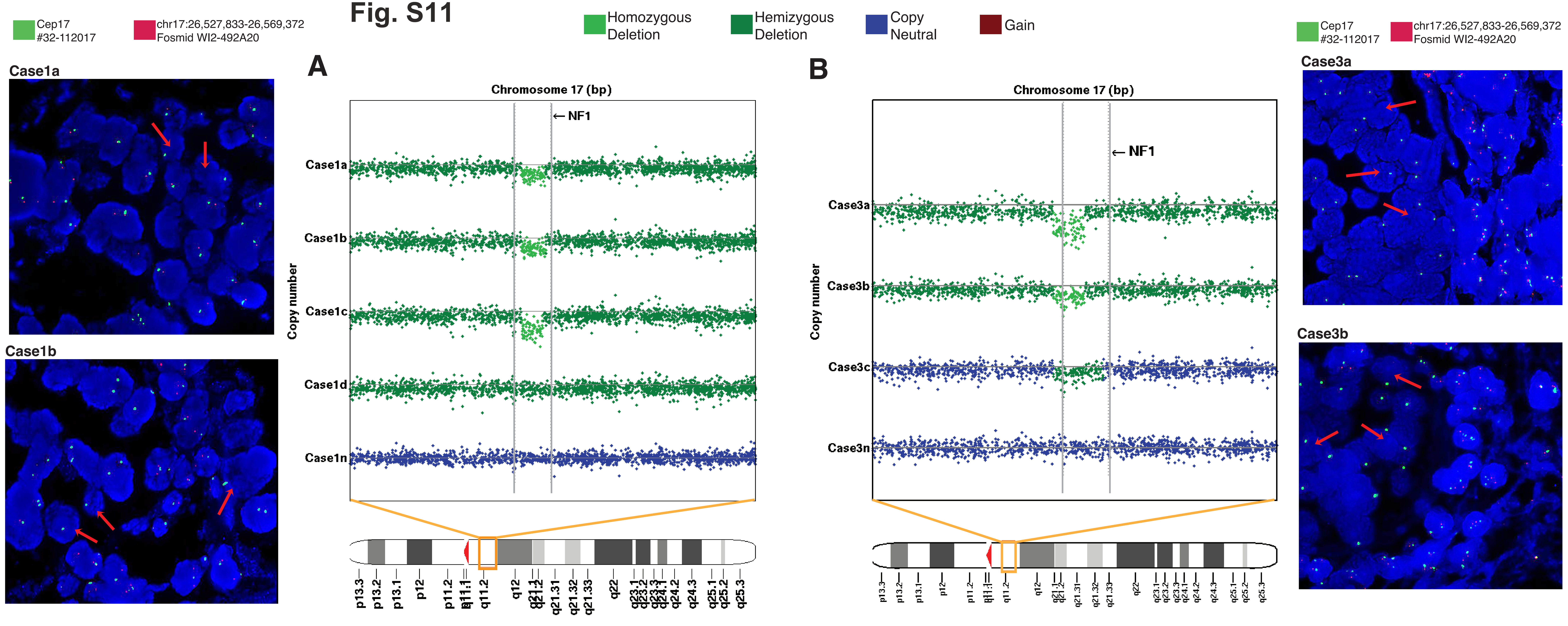

Cep17 #32-112017
chr17:26,527,833-26,569,372 Fosmid WI2-492A20
Homozygous Deletion
Hemizygous Deletion
Copy Neutral
Gain
Case1a
Case1b
A
Chromosome 17 (bp)
← NF1
Case1a
Case1b
Case1c
Case1d
Case1n
Copy number
p13.3
p13.2
p13.1
p12
p11.2
q11.2
q12
q21.31
q21.32
q21.33
q22
q24.2
q24.3
q25.1
q25.2
q25.3
B
Chromosome 17 (bp)
← NF1
Case3a
Case3b
Case3c
Case3n
Copy number
Cep17 #32-112017
chr17:26,527,833-26,569,372 Fosmid WI2-492A20
Case3a
Case3b
