# Supplementary material for: Distinct evolutionary trajectories of primary high-grade serous ovarian cancers revealed through spatial mutational profiling
Source: J Pathol. 2013 Aug 6;231(1):21–34. doi: 10.1002/path.4230 (PMC3864404; doi:10.1002/path.4230)
Supplement: Supplementary file 13 — Figure S13. The pyClone model shown as a probabilistic graphical model. [file path0231-0021-sd13.pdf]

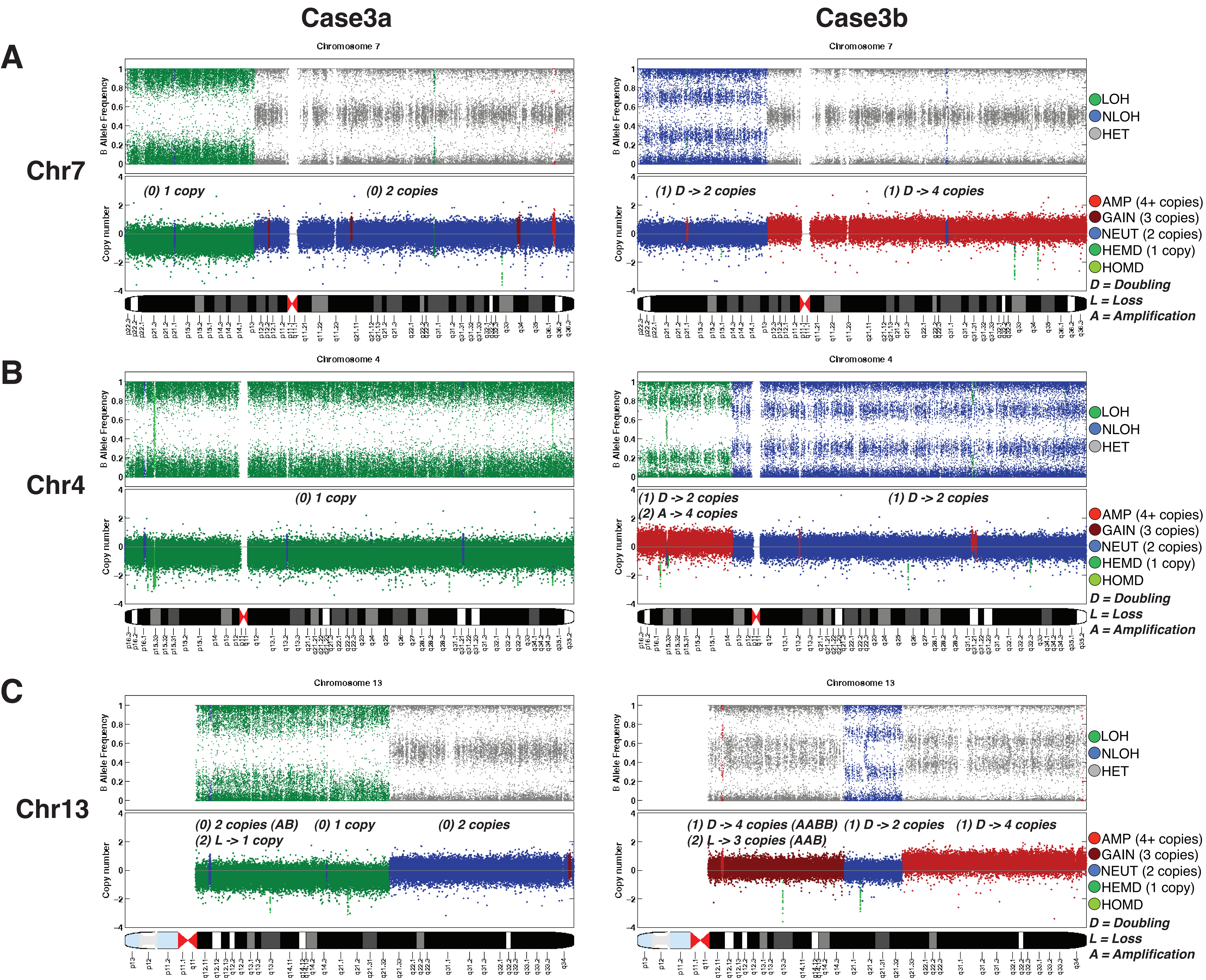

Fig. S12

Competing Explanations for 13q12.11 - 13q14.2

Concurrent Segmental Aneuploidy	Subclonal Segmental Aneuploidy
Case3a: 2CN -> L -> 1CN	Case3a(clone1): 1CN
Case3b: 2CN -> D -> 4CN -> L -> 3CN	Case3a(clone2): 2CN -> L -> 1CN
	Case3b: 2CN(clone2) -> D -> 4CN -> L -> 3CN
