# Supplementary material for: Distinct evolutionary trajectories of primary high-grade serous ovarian cancers revealed through spatial mutational profiling
Source: J Pathol. 2013 Aug 6;231(1):21–34. doi: 10.1002/path.4230 (PMC3864404; doi:10.1002/path.4230)
Supplement: Supplementary file 14 — Table S1. Median and standard deviation of coverage for the exons for the 25 (19 tumour, six normal samples) exome capture libraries. [file path0231-0021-sd14.pdf]

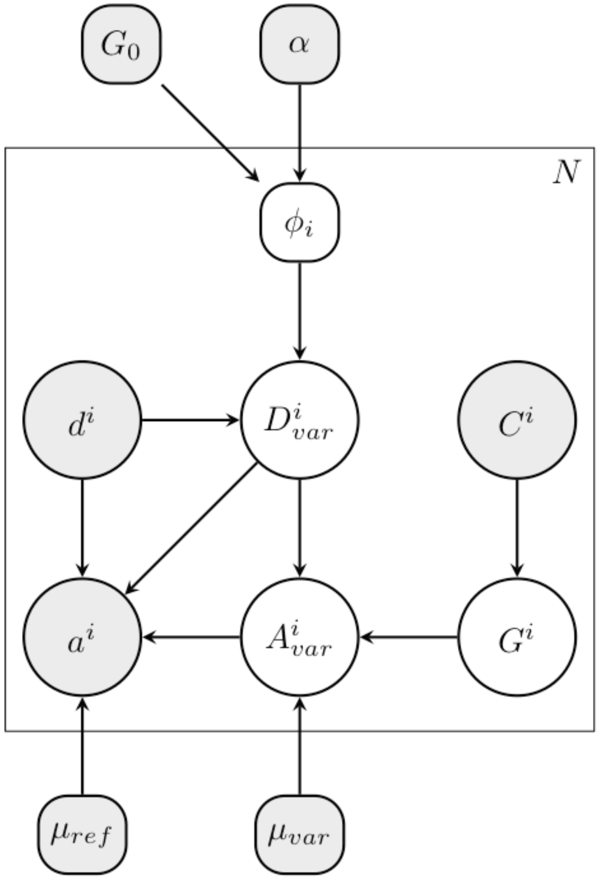

$$
\begin{aligned}
\phi^i &\sim DP(\alpha, G_0) \\
&= \text{Clonal frequency} \\
D^i_{var} | \phi^i, d^i &\sim Binomial(d^i_{var} | \phi^i, d^i) \\
&= \text{Number of reads sampled from clones} \\
A^i_{var} | G^i, \mu_{var}, D^i_{var} &\sim Binomial(a^i_{var} | \mu_{var:cg}, d^i_{var}) \\
&= \text{Number of matches to ref from clones} \\
G^i | C^i &\sim Discrete\,Uniform(g | 1, c) \\
&= \text{Genotype of clone} \\
a^i | A^i_{var}, d^i, D^i_{var}, \mu_{ref} &\sim Binomial(a^i - a^i_{var} | \mu_{ref}, d^i - d^i_{var}) \\
&= \text{Number of matches to ref}
\end{aligned}
$$

Fig. S13
